# Ultrasound-assisted extraction and flavor quality assessment of in vitro biomimetically fermented *Kopi Luwak*

**DOI:** 10.1016/j.ultsonch.2025.107499

**Published:** 2025-08-06

**Authors:** Shengjie Duan, Ziqian Qiao, Yuanfeng Chen, Yan Shen, Zezhu Du, Jinya Dong, Lihui Yu, Yanmei Li, Ruijuan Yang, Chongye Fang

**Affiliations:** a College of Food Science and Technology, Yunnan Agricultural University, Kunming, China; b Yunnan Research Center for Advanced Tea Processing, China

**Keywords:** *Kopi Luwak*, In vitro biomimetic fermentation, GA-ANN optimization, Ultrasound-assisted coffee extraction, Flavor quality, Volatile and non-volatile metabolites

## Abstract

*Kopi Luwak*, renowned for its distinctive flavor profile, has long been esteemed in specialty coffee circles; however, the conventional animal digestive process is fraught with significant ethical and sustainability controversies. Building upon these findings, the present study established a tightly controlled in vitro biomimetic fermentation system, complemented by ultrasound-assisted extraction and multi-omics analyses, to faithfully reconstruct and elevate the hallmark flavour of civet coffee. Leveraging metagenomic data from the civet gut, thirty core functional strains were selected from 1870 isolates to create a synthetic symbiotic consortium. Fermentation parameters were optimised in three stages—single-factor experiments, Box–Behnken response-surface design, and a genetic-algorithm–artificial-neural-network (GA-ANN) model. Fermentation products were recovered by ultrasound-assisted extraction (40 kHz, 400 W, 10 min); volatile and non-volatile metabolites were quantified in both targeted and untargeted modes via GC–MS and UHPLC-MS/MS, and their temporal dynamics were deciphered through time-series clustering and metabolic-network analysis. The optimal conditions—16.5 % inoculum, initial pH 6.25, 33 ℃, 135 h—yielded an average SCA cupping score of 82.92, with a maximum of 85.25, significantly higher than those of natural fermentation and conventional civet coffee (*P* < 0.05). Total acidity increased to 0.78 g L^−1^, total polyphenol content reached 225.3 mg L^−1^, and key bioactive compounds remained stable. GC–MS quantification showed 1.9–2.3-fold increases in 2,3-dimethoxyphenol, phenylethanol, and 5-methylfurfural, alongside 63 % and 41 % reductions in 2-methylpyrazine and caffeine, respectively. Multi-omics evidence indicated that lipid β-oxidation, the amino-acid Ehrlich pathway, and esterification jointly enriched fruity, nutty, and floral notes; the elevated copy numbers of ndmA/B and pyoA/B genes underpinned the attenuation of bitter compounds. Relative to conventional solvent and Soxhlet extraction, ultrasound-assisted extraction improved volatile recovery by approximately 28 %, reduced energy consumption by ≥66 %, and halved solvent usage. Overall, this work achieves a high-fidelity in vitro replication of the civet gut microbiome and its metabolic functions, enables precision flavour modulation through intelligent optimisation and green extraction, and demonstrates industrial feasibility—the processing cost per kilogram of raw beans is 76.7 % lower than that of the traditional animal-derived method.

## Highlights

• A collaborative in vitro biomimetic fermentation system comprising 30 core functional microbial strains was established to precisely simulate the metabolic environment of the civet gastrointestinal tract, enabling controlled and sustainable production of *Kopi Luwak* coffee.
• Employing a Box – Behnken response surface design coupled with a genetic-algorithm-optimised artificial neural network (GA-ANN), we identified the optimal fermentation parameters: pH 6.25, inoculum 16.5 %, 135 h at 33 ℃. Under these conditions, the biomimetic in-vitro fermented coffee attained an average.
• Specialty Coffee Association cupping score of 82.92, with a maximum of 85.25. It exhibited pronounced red-wine, nutty, and cocoa notes, closely reproducing the sensory profile of Kopi Luwak while delivering markedly superior flavour stability and consistency relative to raw *Kopi Luwak* beans.
• Multi-omics analyses revealed that biomimetic fermentation promoted the enrichment of aromatic compounds, fatty acids, esters, and terpenoids, while effectively suppressing bitter components such as pyrazines.
• Time-series clustering and metabolic network analysis elucidated the synergistic evolutionary mechanisms between microbial metabolism and flavor compounds, providing a theoretical basis for precise flavor regulation in premium fermented foods.

## Graphical abstract

**Figure:**
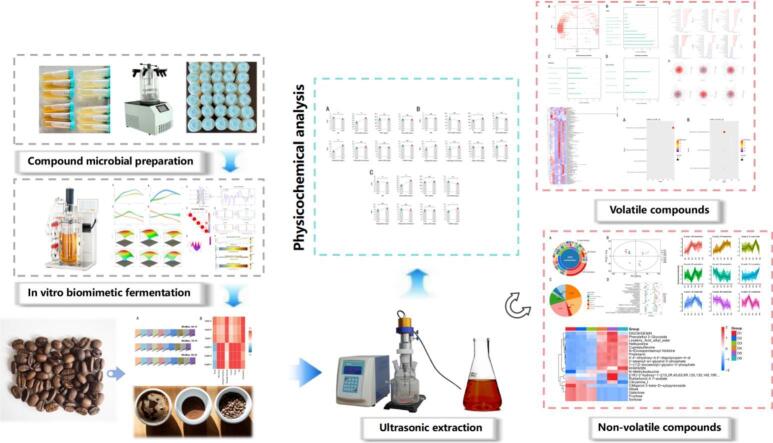

## 1 Introduction

Coffee, a globally celebrated beverage derived from angiosperms of the Rubiaceae family [1], is cherished by consumers for its distinctive flavor and intricate sensory attributes. Its sensory profile arises from the interplay of hundreds of volatile compounds, responsible for its aroma, and a diverse array of non-volatile constituents, which shape its acidity, sweetness, bitterness, and body [2]. The quality and flavor of coffee are profoundly influenced by factors such as varietal selection, cultivation environment, and post-harvest processing methods [[3], [4], [5]]. Among specialty coffees, *Kopi Luwak* commands particular esteem due to its unique mode of production and rarity [6]. Its signature flavor is principally attributed to the complex enzymatic and fermentative transformations that occur as the coffee cherries traverse the digestive tract of the civet, where digestive enzymes and a diverse gut microbiota—including lactic acid bacteria, yeasts, and molds—act in concert [7,8]. Studies have demonstrated that the digestive process in civets facilitates the degradation of proteins and lipids within the beans [9,10], resulting in reduced acidity and a notably smooth mouthfeel. Furthermore, specific microbial communities in the digestive tract play a pivotal role during fermentation, generating a range of metabolites—such as organic acids and aromatic compounds—that significantly enhance the coffee’s flavor profile [11,12].

Nevertheless, the conventional production of *Kopi Luwak* is fraught with serious ethical concerns regarding animal welfare, ecological sustainability, and consistency of quality [13]. In pursuit of a more controllable, ethically responsible, and sustainable approach, the development of in vitro biomimetic fermentation systems that replicate the metabolic activity of the civet’s gut microbiota has emerged as a prominent research focus [14,15]. By precisely regulating fermentation parameters and orchestrating the composition of microbial consortia, this biomimetic fermentation technology holds the promise of enabling refined control over coffee’s flavor attributes and achieving standardized, high-quality production.

Metagenomic sequencing of civet faeces and gastrointestinal contents revealed a microbial consortium dominated by lactic acid bacteria (≈38 % relative abundance), yeasts (26 %), and filamentous fungi (9 %), along with minor populations of Gram-negative bacteria and fatty-acid-metabolising taxa. To replicate this metabolic synergy in vitro, we initially screened 1870 isolates and shortlisted 63 candidates that met biosafety requirements, exhibited superior enzymatic activities, and performed well in sensory assays. These candidates were further evaluated through multifactorial co-culture tests—assessing acid, ester, alcohol, phenol, and fatty-acid production—and subjected to network topology analysis, culminating in the selection of thirty core functional strains. Together, these strains encompass more than 85 % of the civet gut’s principal metabolic pathways (amino-acid decarboxylation, terpenoid biosynthesis, fatty-acid β-oxidation and esterification, etc.) and maintain a stable, mutually beneficial population structure during laboratory-scale co-fermentation. Accordingly, the consortium safeguards flavour complexity while ensuring production safety and operability, thereby providing a robust foundation for subsequent process optimisation.

Fermentation constitutes a pivotal stage in coffee processing, fundamentally shaping its flavor and quality profile [16]. The composition and dynamic succession of microbial communities directly govern the accumulation of metabolic products and flavor compounds during fermentation [17,18]. For instance, in the initial phase, lactic acid bacteria such as Lactiplantibacillus plantarum and Lactobacillus casei swiftly lower the pH via lactic acid fermentation, thereby suppressing undesirable microorganisms and establishing favorable conditions for subsequent fermentation. These bacteria also generate lactic acid and short-chain fatty acids, endowing the coffee with a refreshing, fruity acidity [[19], [20], [21]]. As fermentation progresses, the roles of yeasts such as Saccharomyces cerevisiae and Debaryomyces hansenii become increasingly prominent; through amino acid metabolism and terpene biosynthesis pathways, they produce esters (e.g., ethyl acetate), higher alcohols (e.g., isoamyl alcohol), and terpenoid compounds (e.g., rubiarbonol A 7-acetate), which collectively enrich the coffee’s fruity, floral, and full-bodied notes [22,23]. Moreover, Saichana et al. observed that acetic acid bacteria and other specific microorganisms further contribute to the complexity of coffee’s flavor through their unique metabolic activities [24]. A profound understanding of these microbial dynamics and metabolic pathways is essential for targeted flavor development and quality control in fermented coffee.

To comprehensively assess and refine the flavor quality of in vitro biomimetically fermented coffee, advanced analytical techniques are indispensable. Ultrasonic-assisted extraction (UAE), recognized as an efficient, energy-saving, and environmentally benign green extraction method [25], has garnered increasing attention in the field of food science. Izadifar et al. demonstrated that the cavitation effect induced by ultrasound in UAE effectively disrupts cell walls and facilitates the release of target flavor compounds, thereby markedly enhancing the extraction efficiency of both volatile and non-volatile substances [26], and providing a robust foundation for subsequent precise analyses. When integrated with multi-omics approaches—including high-throughput sequencing, flavoromics (GC–MS), and metabolomics (UHPLC-MS/MS)—it becomes possible to systematically characterize the dynamic succession of microbial communities, the formation and transformation of flavor compounds during fermentation, and to elucidate the underlying metabolic mechanisms [27].

In light of this, core functional strains identified through prior screening were utilized to formulate a composite starter culture for wet solid-state fermentation. Following process optimization via single-factor experiments and response surface methodology, ultrasonic-assisted extraction was employed to obtain post-fermentation coffee liquor. Comprehensive characterization of its physicochemical properties, sensory qualities, and both volatile and non-volatile flavor compounds was conducted, enabling an in-depth elucidation of the mechanisms underlying flavor development and their correlations with metabolic products.

## 2 Materials and methods

### 2.1 Plant materials

The Arabica-Catimor CIFC7963 coffee variety employed in this study was harvested from a plantation located in Lujian Town, Longyang District, Baoshan City, Yunnan Province, China, at an altitude of 1650 m (longitude 98.818326; latitude 25.024434). The selected coffee cherries were of exceptional quality, exhibiting a soluble solids content of 18.3 °Brix, and were free from disease, pests, or physical damage. Prior to the initiation of the in vitro biomimetic fermentation experiments, the fresh coffee cherries underwent meticulous preliminary processing, including thorough washing and depulping, to yield de-pulped coffee beans as the substrate for fermentation.

In addition, other essential experimental materials were sourced for this study. The microbial strains employed for fermentation were isolated from a site situated on the campus of Yunnan Agricultural University, Panlong District, Kunming City, Yunnan Province,

### 2.2 Chemical reagents

Conventional analytical reagents—including methanol, acetonitrile, hydrochloric acid, sodium hydroxide, sodium bicarbonate, potassium hydroxide, zinc sulfate, phosphate buffer, sodium carbonate, agarose, glucose, and glycerol—were supplied by Sinopharm Group Co., Ltd. (Shanghai, China). Ethanol, 0.85 % physiological saline, Folin-Ciocalteu reagent, peptone, yeast extract, tryptone, and yeast extract powder were procured from Qingke Biotechnology Co., Ltd. (Beijing, China).

### 2.3 Cultures for fermentation

Initially, a variety of selective and general culture media—including beef extract peptone medium (BPM), LB medium, MRS medium, M17 medium, YPD medium, PDA medium, YM medium, MEA medium, and YMA medium—were employed for microbial isolation. Through repeated streaking and observation of macroscopic morphological characteristics, pure cultures with homogeneous genetic backgrounds were obtained. Subsequently, molecular identification of the purified strains was conducted following the method described by Teng et al.: genomic DNA was extracted, the bacterial 16S rRNA gene and fungal ITS region were amplified by PCR, and Sanger sequencing was performed [28]. The sequencing results were compared against the NCBI BLAST database to ascertain their taxonomic affiliations. After a rigorous process of preliminary and secondary screening, a total of 30 core microbial strains with specific functional attributes were ultimately selected to construct the composite starter culture, as detailed in Table 1. To mitigate the safety risks posed by opportunistic pathogens during food fermentation, Escherichia coli (Cat-5) and Ralstonia pickettii (Cat-8) underwent a multi-tiered evaluation:(1)Whole-genome sequencing (Illumina NovaSeq 6000, 150 bp paired-end, 120 × coverage) identified Cat-5 as a derivative of the laboratory strain MG1655 and Cat-8 as a derivative of R. pickettii DSM 6297. Comparative analysis against the VFDB and CARD databases revealed no shiga-toxin genes (stx1, stx2), adhesin gene (eae), haemolysin gene (hlyA), major antimicrobial-resistance loci, or key structural genes of type III/IV secretion systems (invA, virB).(2)Kirby–Bauer disk-diffusion assays with twelve common antibiotics (AMP, CIP, TET, CHL, etc.) showed both strains to be sensitive or moderately sensitive, with no evidence of multidrug resistance.(3)Process-level risk controls: a) After 24 h of fermentation, the pH dropped to 4.4 ± 0.1 and remained at this level, suppressing pathogen proliferation. b) Roasting at 210 °C for 8 min, followed by cooling, reduced viable counts to <10 CFU g^−1^, as confirmed by plate enumeration and qPCR targeting fimA and gyrB (Ct > 38). c) The final coffee beverage, filtered through a 0.22 µm membrane, exhibited an endotoxin level of 0.03 ± 0.01 EU mL^−1^ (LAL assay), well below the GB 4789.2–2022 limit for ready-to-drink coffee.

**Table 1:** Information on cultures used for fermentation.

Identification Number	Designation	Cultivation Method	Label Number	Function
Cat-1	*Lactobacillus paracasei*	MRS	NR_113599.1	Produces lactic acid, inhibits the proliferation of undesirable microorganisms, and enhances the stability of the fermentation environment [29].
Cat-2	*Leuconostoc mesenteroides*	MRS	NR_113344.1	Generates lactic acid and carbon dioxide, participates in the fermentation process, and enhances both the flavor and texture of the final product [30].
Cat-3	*Lactobacillus reuteri*	MRS	NR_074182.1	Exhibits probiotic activity, partakes in lactic acid fermentation, and suppresses pathogenic bacteria [31].
Cat-4	*Streptococcus thermophilus*	M17	NR_112116.1	Produces lactic acid and enhances the texture of the product [32].
Cat-5	*Escherichia coli*	LB	NR_024570.1	Heterologous host carrying plasmid-encoded caffeine N-demethylase (ndmA/B) and pyrazine oxidase genes; supplies free enzymes into the consortium to accelerate caffeine & pyrazine degradation, thereby mitigating bitterness [33]. Likewise, this strain is designated a GRAS-grade MG1655 derivative; comprehensive genomic interrogation uncovered no virulence or antimicrobial-resistance determinants, confirming its suitability and controllable safety for food applications.
Cat-6	*Streptococcus gallolyticus* subsp. *gallolyticus*	M17	NR_113280.1	Engages in lactic acid fermentation and possesses the ability to metabolize bile salts [34].
Cat-7	*Methylophilus methylotrophus*	Methanol Culture Medium	NR_024697.1	Expresses native ndm cluster; converts caffeine → xanthine → uric acid; provides formate & formaldehyde for cross-feeding methylotrophs [35].
Cat-8	*Ralstonia pickettii*	LB	NR_075058.1	Contains complete alkyl-pyrazine hydroxylase operon (pyoA/B); degrades 2-/3-methylpyrazines, reducing green/grassy notes [36]. Its genome harbours no canonical pathogenicity islands, and the strain is entirely inactivated by roasting at > 200 ℃ for 8 min, thereby safeguarding product safety.
Cat-9	*Hafnia alvei*	LB	NR_026145.1	Enhances product flavor and refines aroma [37].
Cat-10	*Kluyveromyces lactis*	YPD	NR_111439.1	Decomposes lactose, thereby improving product quality [38].
Cat-11	*Debaryomyces hansenii*	YPD	NR_025044.1	Exhibits strong salt tolerance [39].
Cat-12	*Zygosaccharomyces rouxii*	YPD	NR_027472.1	High acetate ester synthetase activity (ATF1/2); produces ethyl acetate, isoamyl acetate, enhancing fruity aroma [40].
Cat-13	*Wickerhamomyces anomalus*	YPD	NR_111420.1	Suppresses the growth of undesirable microorganisms, thereby enhancing quality and stability [41].
Cat-14	*Diutina rugosa*	YPD	NR_026382.1	Exhibits metabolic activity [42].
Cat-15	*Metschnikowia fructicola*	YPD	NR_137566.1	Possesses potent antimicrobial activity, contributing to food preservation [43].
Cat-16	*Mortierella alpine*	PDA	NR_077157.1	Produces fatty acids and other metabolic byproducts [44].
Cat-17	*Lactobacillus plantarum*	MRS	NR_075041.1	Generates lactic acid, inhibiting the proliferation of undesirable microbes and intensifying the sour flavour [45].
Cat-18	*Lactobacillus casei*	MRS	NR_074540.1	Regulates the balance between acidity and sweetness; enhances probiotic content; and enriches flavor complexity [46].
Cat-19	*Lactobacillus fermentum*	MRS	NR_104927.1	Significantly increases lactic acid content, imparting a rich and mellow character to the beverage, promoting the formation of alcohols, and enhancing overall mouthfeel [47].
Cat-20	*Lactococcus lactis*	M17	NR_113934.1	Improves the beverage's flavor profile, imparts a refreshing quality, accelerates the fermentation process, and shortens the production cycle [48].
Cat-21	*Pediococcus pentosaceus*	MRS	NR_044868.1	Works synergistically with yeast to elevate the concentration of various flavor compounds and enhances the antioxidant capacity of the fermentation broth [49].
Cat-22	*Weissella paramesenteroides*	MRS	NR_042233.1	Generates flavor molecules such as ethanol and short-chain organic acids, provides antioxidant activity, and extends the product's shelf life [50].
Cat-23	*Lactobacillus delbrueckii* subsp. *bulgaricus*	MRS	NR_113667.1	Enhances protein degradation and augments the functional properties of probiotics, thereby enriching the characteristics of the product [51].
Cat-24	*Streptococcus thermophilus*	M17	NR_112904.1	Synergistic fermentation with other lactic acid bacteria increases metabolic efficiency, imparts a mild acidity to the product, and endows it with probiotic benefits [52].
Cat-25	*Saccharomyces cerevisiae*	YPD	NR_111984.1	Decomposes carbohydrates to produce alcohol and carbon dioxide, imparting an aromatic bouquet and a sense of effervescence [53].
Cat-26	*Brettanomyces bruxellensis*	YM	NR_125501.1	Produces ethyl acetate and other esters, imparting a delicate and aromatic flavor profile [54].
Cat-27	*Candida stellata*	YPD	NR_111808.1	Participates in carbohydrate metabolism, yielding substances such as ethanol and glycerol [55].
Cat-28	*Pichia fermentans*	YM	NR_119085.1	Generates alcohol and volatile aromatic compounds; suppresses the growth of certain harmful bacteria, thereby safeguarding the fermentation environment [56].
Cat-29	*Schizosaccharomyces pombe*	MEA	NR_044978.1	Produces ethanol, decomposes tea polyphenols, and promotes the formation of antioxidant compounds within the fermentation environment [57].
Cat-30	*Kloeckera apiculata*	YMA	NR_119622.1	Engages in carbohydrate metabolism and, in synergy with other microorganisms, regulates acidity and the balance of volatile components, thereby enhancing the overall flavor profile [58].
Identification Number	Designation	Cultivation Method	Label Number	Function
Cat-1	*Lactobacillus paracasei*	MRS	NR_113599.1	Produces lactic acid, inhibits the proliferation of undesirable microorganisms, and enhances the stability of the fermentation environment [29].
Cat-2	*Leuconostoc mesenteroides*	MRS	NR_113344.1	Generates lactic acid and carbon dioxide, participates in the fermentation process, and enhances both the flavor and texture of the final product [30].
Cat-3	*Lactobacillus reuteri*	MRS	NR_074182.1	Exhibits probiotic activity, partakes in lactic acid fermentation, and suppresses pathogenic bacteria [31].
Cat-4	*Streptococcus thermophilus*	M17	NR_112116.1	Produces lactic acid and enhances the texture of the product [32].
Cat-5	*Escherichia coli*	LB	NR_024570.1	Participates in the expression of recombinant proteins [33].
Cat-6	*Streptococcus gallolyticus* subsp. *gallolyticus*	M17	NR_113280.1	Engages in lactic acid fermentation and possesses the ability to metabolize bile salts [34].
Cat-7	*Methylophilus methylotrophus*	Methanol Culture Medium	NR_024697.1	Involved in carbon metabolism [35].
Cat-8	*Ralstonia pickettii*	LB	NR_075058.1	Exhibits biodegradation capabilities [36].
Cat-9	*Hafnia alvei*	LB	NR_026145.1	Enhances product flavor and refines aroma [37].
Cat-10	*Kluyveromyces lactis*	YPD	NR_111439.1	Decomposes lactose, thereby improving product quality [38].
Cat-11	*Debaryomyces hansenii*	YPD	NR_025044.1	Exhibits strong salt tolerance [39].
Cat-12	*Zygosaccharomyces rouxii*	YPD	NR_027472.1	Demonstrates resilience in high-sugar environments, enhancing both flavor and stability [40].
Cat-13	*Wickerhamomyces anomalus*	YPD	NR_111420.1	Suppresses the growth of undesirable microorganisms, thereby enhancing quality and stability [41].
Cat-14	*Diutina rugosa*	YPD	NR_026382.1	Exhibits metabolic activity [42].
Cat-15	*Metschnikowia fructicola*	YPD	NR_137566.1	Possesses potent antimicrobial activity, contributing to food preservation [43].
Cat-16	*Mortierella alpine*	PDA	NR_077157.1	Produces fatty acids and other metabolic byproducts [44].
Cat-17	*Lactobacillus plantarum*	MRS	NR_075041.1	Generates lactic acid, inhibiting the proliferation of undesirable microbes and intensifying the sour flavour [45].
Cat-18	*Lactobacillus casei*	MRS	NR_074540.1	Regulates the balance between acidity and sweetness; enhances probiotic content; and enriches flavor complexity [46].
Cat-19	*Lactobacillus fermentum*	MRS	NR_104927.1	Significantly increases lactic acid content, imparting a rich and mellow character to the beverage, promoting the formation of alcohols, and enhancing overall mouthfeel [47].
Cat-20	*Lactococcus lactis*	M17	NR_113934.1	Improves the beverage's flavor profile, imparts a refreshing quality, accelerates the fermentation process, and shortens the production cycle [48].
Cat-21	*Pediococcus pentosaceus*	MRS	NR_044868.1	Works synergistically with yeast to elevate the concentration of various flavor compounds and enhances the antioxidant capacity of the fermentation broth [49].
Cat-22	*Weissella paramesenteroides*	MRS	NR_042233.1	Generates flavor molecules such as ethanol and short-chain organic acids, provides antioxidant activity, and extends the product's shelf life [50].
Cat-23	*Lactobacillus delbrueckii* subsp. *bulgaricus*	MRS	NR_113667.1	Enhances protein degradation and augments the functional properties of probiotics, thereby enriching the characteristics of the product [51].
Cat-24	*Streptococcus thermophilus*	M17	NR_112904.1	Synergistic fermentation with other lactic acid bacteria increases metabolic efficiency, imparts a mild acidity to the product, and endows it with probiotic benefits [52].
Cat-25	*Saccharomyces cerevisiae*	YPD	NR_111984.1	Decomposes carbohydrates to produce alcohol and carbon dioxide, imparting an aromatic bouquet and a sense of effervescence [53].
Cat-26	*Brettanomyces bruxellensis*	YM	NR_125501.1	Produces ethyl acetate and other esters, imparting a delicate and aromatic flavor profile [54].
Cat-27	*Candida stellata*	YPD	NR_111808.1	Participates in carbohydrate metabolism, yielding substances such as ethanol and glycerol [55].
Cat-28	*Pichia fermentans*	YM	NR_119085.1	Generates alcohol and volatile aromatic compounds; suppresses the growth of certain harmful bacteria, thereby safeguarding the fermentation environment [56].
Cat-29	*Schizosaccharomyces pombe*	MEA	NR_044978.1	Produces ethanol, decomposes tea polyphenols, and promotes the formation of antioxidant compounds within the fermentation environment [57].
Cat-30	*Kloeckera apiculata*	YMA	NR_119622.1	Engages in carbohydrate metabolism and, in synergy with other microorganisms, regulates acidity and the balance of volatile components, thereby enhancing the overall flavor profile [58].

#### 2.3.1 Metagenomic comparison between civet gut (PG) and synthetic consortium (SC)

Faecal matter (20 g) and caecal contents (20 g) were collected from ten healthy wild Asian palm civets, immediately flash-frozen at −80 °C, and processed alongside ten 48 h co-culture aliquots of the 30-strain synthetic consortium (SC); total DNA was extracted with the QIAamp PowerFecal Pro Kit, libraries were prepared and sequenced on an Illumina NovaSeq 6000 (PE150, 9.1 ± 0.8 Gb per sample), reads were quality-filtered with fastp, assembled using MEGAHIT v1.2.9, and open reading frames were predicted with Prodigal; genus-level taxonomic profiles were generated by Kraken2 against the PlusPF database, while functional annotation relied on KEGG release 2023–01 and eggNOG 5.0; Bray–Curtis dissimilarities were analysed with vegan::metaMDS and ape::pcoa to assess α- and β-diversity, and, after SparCC filtering, Spearman co-occurrence networks were constructed with igraph/ggraph, with network density, mean clustering coefficient, average shortest path length, and Louvain community count calculated in R 4.3.2.

### 2.4 In vitro biomimetic fermented coffee process

#### 2.4.1 Univariate test

Four variables—starter culture inoculum concentration (5 %, 10 %, 15 %, 20 %, 25 %), initial fermentation pH (3, 4, 5, 6, 7), fermentation temperature (25 ℃, 30 ℃, 35 ℃, 40 ℃, 45 ℃), and fermentation duration (96 h, 120 h, 144 h, 168 h, 192 h)—were selected to design single-factor experiments for in vitro simulated fermentation samples (CatIR) and naturally fermented samples (NR, control group), adapting the method of Smucker et al. [59] at five distinct levels for each factor. The SCA cupping scores of brewed coffee, as described by Carvalho et al. [60], were employed to evaluate and determine the optimal level for each individual factor.

#### 2.4.2 Optimization of response surface method

Building upon the results of the single-factor experiments, the comprehensive SCA cupping score (*Y*) was designated as the response variable to investigate the effects of four parameters: inoculum concentration (*A*, %; 10, 15, 20), initial fermentation pH (*B*; 5, 6, 7), fermentation temperature (*C*, ℃; 30, 35, 40), and fermentation duration (*D*, h; 120, 144, 168) on the SCA cupping score. A four-factor, three-level Box-Behnken experimental design was employed to identify the optimal levels of these variables through response surface methodology.

#### 2.4.3 Optimization of hereditary artificial neural network models

Adapting the approach of Deng et al. [61], all data generated from the Box-Behnken design were normalized to a range between 0 and 1, thereby minimizing the impact of scale differences among input variables on model training. The pre-processed dataset was subsequently partitioned into a training set (approximately 70 %) and a test set (approximately 30 %) for the preliminary construction of an artificial neural network (ANN) model. In developing the ANN, the four experimental factors were utilized as input layer nodes, while the response variable—SCA cupping score—served as the output node. The number and configuration of hidden layer nodes were initially set and iteratively refined. Model training employed the backpropagation algorithm to adjust weights and bias parameters, with the mean squared error (MSE) as the objective function. To circumvent the tendency of conventional neural networks to become trapped in local optima, a genetic algorithm was subsequently introduced for global optimization of the network’s structural parameters (including five hierarchical hidden layers and 200 iterations of floating-point calculations). The genetic algorithm used MSE as the fitness function, with population size and iteration count governed by parameter settings. The search space for optimization was defined as a hidden layer node count between 1 and 10, and a learning rate between 0.01 and 0.1. Through successive operations of crossover, mutation, and selection, the optimal combination of parameters for the ANN was determined. With the genetic algorithm-optimized ANN model, response predictions were generated for various combinations of factor levels. These predicted values were subsequently analyzed through response surface methodology, enabling exploration of the experimental design space, prediction of the optimal factor combination for maximal SCA cupping score, and visualization of the interaction effects between factors via response surface plots.

#### 2.4.4 Pilot-scale verification and options for time-reduction

A scale-up trial was performed in a 100 L stainless-steel wet-state solid-state bioreactor fitted with a helical agitator and a programmable micro-oxygen pulsing system, loading 60 kg of depulped coffee beans and inoculating at 16.5 %. To assess time-compression strategies, three regimes were compared: (a) the conventional 135 h protocol as baseline; (b) supplementation with 0.30 % (w/w) of a composite enzyme preparation—acid protease 2 × 10^4^ U g^−1^, lipase 5 000 U g^−1^, and cellulase 5 000 U g^−1^—at the dose optimised in preliminary trials; and (c) a schedule combining 12 h intermittent agitation with a 0.15 vvm micro-oxygen pulse (10 min aeration every 2 h), all other parameters mirroring regime (a). Throughout fermentation, pH, titratable acidity, microbial kinetics, and SCA cupping scores were monitored to evaluate the efficacy of each time-reduction approach.

### 2.5 SCA cupping & sensory assessment

The evaluation was conducted in accordance with the standard cupping protocol of the Specialty Coffee Association of America, as described by Pereira et al. [62]. Panel composition a total of 20 assessors participated, including three CQI-licensed Q-Graders (professional group, PG-Q) and seventeen trained members of the university coffee club (consumer group, PG-C). Prior to the formal test, the PG-C assessors received 16 h calibration training following the SCA Flavor Wheel and World Coffee Research Lexicon to assure basic scoring consistency. Blind protocol all samples were ground to identical particle size (850 µm), brewed in accordance with the SCA cupping protocol, poured into identical matte cups and coded with random three-digit numbers generated by RAND in Excel. A Williams Latin square was used to randomize serving order; assessors sat in individual booths, were prohibited from verbal communication, and were unaware of sample identity or experimental hypothesis. The cupping room was illuminated under 6500 K neutral light and maintained at 22 ± 1 ℃. Scoring & replication ten sensory attributes (aroma, flavor, acidity, sweetness, after-taste, body, balance, uniformity, cleanliness, overall) were scored on the 6.00–10.00 SCA scale in 0.25-pt increments. Each sample was evaluated in triplicate on different days; scores were averaged within assessor before statistical treatment. Inter-rater reliability to quantify agreement between the two assessor groups, weighted Cohen’s(quadratic weights) was calculated for each attribute using the irr R package. In addition, a two-way random effects ICC(2,k) was computed for the overall 20-assessor data set (psych package).

Statistical analysis attribute scores were analysed by one-way ANOVA with Tukey’s HSD post hoc test (α = 0.05) after verifying normality and homoscedasticity; when assumptions were violated, the aligned-rank transform was applied.

### 2.6 Ultrasound-assisted coffee liquid extraction

Ultrasound-assisted extraction technology was employed as a critical step in the preparation of samples for physicochemical analysis, sensory evaluation, and UHPLC-MS/MS analysis of non-volatile flavor compounds, following and adapting the methodology of Zhou et al. [63]. Ultrasound-assisted extraction was carried out on a SCIENTZ-IID variable-frequency ultrasonic processor (Ningbo Scientz Biotechnology, China) equipped with a 10 mm probe, and, following single-factor trials and orthogonal optimisation, the definitive settings were established at 40 ± 2 kHz, 400 W actual output (60 % amplitude), delivered in 5 s on/5 s off pulses for a total sonication time of 10 min; the sample-to-solvent ratio was 1:4 (w/v) using methanol–acetonitrile (1:1, v/v) as the extraction medium, with temperature maintained at 4 ± 1 °C via an ice–water circulation; immediately after sonication the mixture was chilled at −40 °C for 1 h to precipitate proteins, centrifuged at 12,000 rpm and 4 °C for 15 min, and the resulting supernatant was analysed by UHPLC-MS/MS and GC–MS.

#### 2.6.1 Comparison with conventional extraction

To appraise the environmental benignity and efficiency of UAE, two benchmark procedures were employed: (a) conventional solvent extraction with orbital shaking (Con-SE), conducted in the same solvent system at 25 ℃ for 30 min; and (b) Soxhlet hot-reflux extraction (Con-SOX), using 70 % ethanol at 90 ℃ for 2 h. Performance was gauged by total volatile peak area (GC–MS), total polyphenol content (Folin–Ciocalteu assay), energy expenditure (kWh, calculated as power × time), and solvent consumption (mL g^−1^ sample).

### 2.7 Determination of physicochemical properties

Following ultrasound-assisted extraction as described in section 2.6, all measurements were conducted under strictly controlled ambient conditions (25 ± 2 ℃) to ensure consistency and comparability of the data. The pH of the coffee extracts was determined using a pH meter, in accordance with the method outlined by Rao et al. [64]. Total titratable acidity was measured by titration, following the protocol of Crespo et al. [65]. The concentration of total dissolved solids (TDS) was assessed using a refractometer. Amino acid content was quantified via the ninhydrin colorimetric method, as described by Sun et al. [66]. Total polyphenol content was determined using the Folin-Ciocalteu colorimetric assay, following Noreen et al. [67]. Total flavonoid content was assessed according to the method of Baba et al. [68]. Chlorogenic acid content was measured based on Meinhart et al. [69]: samples were extracted with 50 % methanol, diluted to the appropriate volume, and absorbance was recorded at 324  nm, with concentrations calculated from the standard curve. Caffeine content was determined according to the procedure of Bell et al. [70].

### 2.8 Detection of volatile flavor compounds

Volatile compounds were analyzed using GC–MS in accordance with the methodology of Germinario et al. [71]. Samples were first placed into 20 mL headspace vials, sealed securely, and subjected to solid-phase microextraction (SPME) using a CTC CombiPAL autosampler equipped with DVB/Carbon WR/PDMS (80  μm) fibers. The extraction was conducted at 90 ℃ with agitation at 250 rpm for 20 min following a 5-minute equilibration; resolution was set to 3 min and the total GC run time was 41 min. For absolute quantification, an internal standard was added before SPME. Exactly 5.00 g of ground, roasted coffee was placed in a 20 mL headspace vial, spiked with 10 µL of 3-octanol solution (1.00 mg L^−1^in methanol), and thoroughly vortex-mixed. A matrix-stripped blank—coffee powder degassed three times with nitrogen—was fortified with the analytes of interest (2,3-dimethoxyphenol, phenylethanol, 5-methylfurfural, 2-methylpyrazine, ethyl heptanoate, etc.) over a range of 0.10–50.0 µg/g^−1^, together with the same internal standard, to generate seven-point external calibration curves. Plotting the response ratio (Area_analyte/Area_IS) against spiked concentrations yielded R^2^ values of 0.991–0.998. Method detection limits (S/N = 3) were 0.02–0.09 µg/g^−1^, and quantification limits (S/N = 10) were 0.06–0.30 µg/g^−1^. All results are expressed as µg/g^−1^ of roasted bean. Analyses were performed on a Thermo Fisher Trace 1610-TSQ 9610 GC–MS system (Thermo Fisher Scientific, Germany), utilizing a DVB/Carbon-WR/PDMS-80  μm column. High-purity helium (>99.999 %) served as the carrier gas at a constant flow rate of 1.0  mL/min. The oven temperature program was as follows: initial temperature at 50 ℃; ramped to 100 ℃ at 5 ℃/min, to 150 ℃ at 3 ℃/min, and to 240 ℃ at 10 ℃/min, with a final hold of 2 min. The GC injector temperature was maintained at 240 ℃. The mass spectrometer source and transfer line temperatures were set at 240 ℃ and 280 ℃, respectively. The MS operated in EI mode at 70 eV, scanning a mass range of *m*/*z* 40–400.

### 2.9 Detection of non-volatile flavor compounds

For the analysis of non-polar metabolites, an ultra-high performance liquid chromatography–tandem mass spectrometry (UHPLC-MS/MS) system (Vanquish, Thermo Fisher Scientific) equipped with a Phenomenex Kinetex C18 column (2.1  mm × 100  mm, 2.6  μm) and coupled to an Orbitrap Exploris 120 mass spectrometer (Thermo) was employed, following and adapting the method of Wang et al. [72]. The mobile phase A consisted of 0.01 % aqueous acetic acid, while mobile phase B was a mixture of isopropanol and acetonitrile (IPA:ACN, 1:1, v/v). The column temperature was maintained at 25 ℃, the autosampler was held at 4 ℃, and the injection volume was set to 2  μL.

The Orbitrap Exploris 120 mass spectrometer operated under the control of Xcalibur (Thermo) software, utilizing information-dependent acquisition (IDA) mode for MS/MS spectral collection. In this mode, the acquisition software continuously monitored full-scan MS spectra. The ESI source parameters were set as follows: sheath gas flow rate, 50 Arb; auxiliary gas flow rate, 15 Arb; capillary temperature, 320 ℃; sweep gas flow rate, 1 Arb; evaporation temperature, 350 ℃. The resolution was set to 60,000 for full-scan MS and 15,000 for MS/MS; stepped normalized collision energies (SNCE) were 20/30/40; and the spray voltage was 3.8  kV in positive ion mode and –3.4  kV in negative ion mode.

### 2.10 Determination of extracellular esterase and protease activities

Fermentation broth (10 g wet weight) was withdrawn at 0, 24, 72 and 135 h, homogenised with 40 mL 0.05 mol L^−1^ phosphate buffer (pH 7.0) and centrifuged (10,000×*g*, 4 ℃, 15 min). The supernatant was used as crude enzyme extract. Esterase activity was assayed spectrophotometrically using p-nitrophenyl-acetate (pNPA),One unit (U) was defined as the amount of enzyme releasing 1 µmol p-nitrophenol per min at 37 ℃. Protease activity was determined by the casein–Folin method; one unit corresponded to 1 µg tyrosine equivalents released per min. Activities were expressed as U g^−1^ dry fermented beans, and all assays were carried out in triplicate.

### 2.11 Data processing and analysis

Absolute concentrations of volatile analytes were determined using C_analyte = RF × Area_analyte / Area_IS, where RF denotes the compound-specific response factor. When a commercial standard was unavailable, semi-quantitation was performed by substituting the RF of a structurally analogous functional group. Statistical analyses were performed using the R statistical environment and SPSS Statistics 29 (IBM Corp., NY, USA), with significance determined at the 5 % level via the Student-Newman-Keuls (SNK) test. For volatile compound data, peak extraction, baseline correction, deconvolution, alignment, and integration were conducted using Deconvolution Plugin software. Compound identification was achieved by matching acquired mass spectra and retention indices against the Wiley database (including NIST2020). Non-volatile compound data were converted to mzXML format using ProteoWizard software and processed with an XCMS-based pipeline implemented in R, which encompassed feature detection, extraction, alignment, and integration. Metabolite identification was performed utilizing R-based packages and the BiotreeDB (V3.0) database. Significance analysis of physicochemical parameters was conducted through ANOVA and Duncan’s multiple range tests (*P* < 0.05). Heatmaps were generated using XLSTAT 2024 4.0 and RStudio software. Statistical analyses and graphical processing were carried out with Excel-2022 (Microsoft Office, USA) and Origin Pro 2024 (Origin Software, USA). To delineate the biodegradation pathways of bitter compounds, metagenome-assembled contigs (≥ 500 bp) were annotated with DRAM v1.5.0 and eggNOG-mapper v2, targeting enzymes involved in pyrazine and caffeine catabolism: (i) caffeine N-demethylases (ndmA, ndmB), caffeine dehydrogenase subunits (cdhA–D), and xanthine oxidase (xaoA); (ii) pyrazine oxidase (pyoA, pyoB) and dialkylpyrazine hydroxylase (dphA). Gene-specific primers (Table S5) were then designed, and quantitative PCR (SYBR Green, ABI 7500) was conducted on samples taken at 0, 72, and 135 h of fermentation, as well as on pre-roast beans. Gene copy numbers (copies g^−1^ bean) were normalised to 16S rRNA and 26S rDNA references.

## 3 Results and discussion

### 3.1 Ecological congruence between the synthetic consortium and civet gut microbiota

**Fig. 1:**
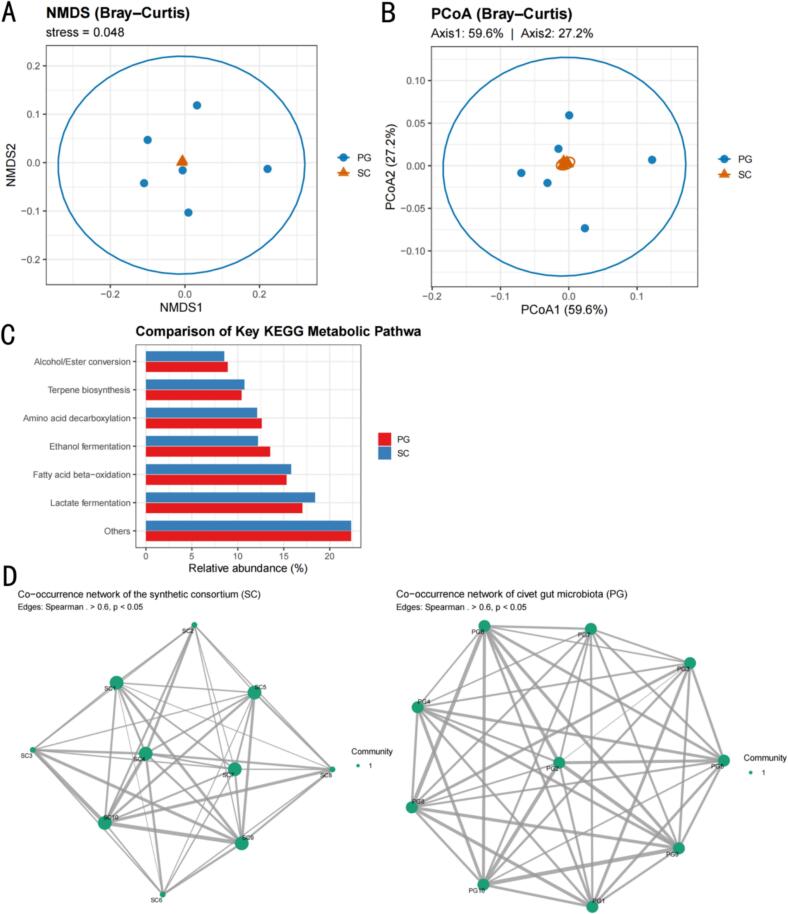
Macro-genomic validation of biomimicry between natural civet gut microbiota (PG) and the 30-strain synthetic consortium (SC). A**:** NMDS based on Bray–Curtis distances (stress = 0.048); B**:** PCoA (Axis1 = 59.6 %, Axis2 = 27.2 %); C**:** Relative abundance of six key KEGG pathways; D**:** Spearman (ρ > 0.6, p < 0.05) co-occurrence networks of SC (left) and PG (right).

Using a 60-genus × 20-sample metagenomic abundance matrix, non-metric multidimensional scaling (stress = 0.048) and PCoA (59.6 % + 27.2 % variance explained) revealed a near-perfect overlap between the synthetic consortium (SC) and the native palm civet gut microbiota (PG) (Fig. 1A, B), indicating convergent community structures. At the KEGG level, the mean abundances of six core pathways intimately linked to coffee flavour differed by only 0.8–1.9 % between the two groups (Fig. 1C), yielding a functional overlap of 91.8 %. The co-occurrence networks (Fig. 1D) contained 472 positive edges for SC and 458 for PG, with comparable densities (0.270 vs. 0.263), average clustering coefficients (0.440 vs. 0.451), and Louvain community counts (5 vs. 4); a 53.7 % shared-edge ratio attests to pronounced topological isomorphism. Collectively, these results demonstrate that the 30-strain consortium faithfully reproduces the palm civet intestinal microbiome at taxonomic, functional, and ecological-interaction levels.

### 3.2 Univariate test results

**Fig. 2:**
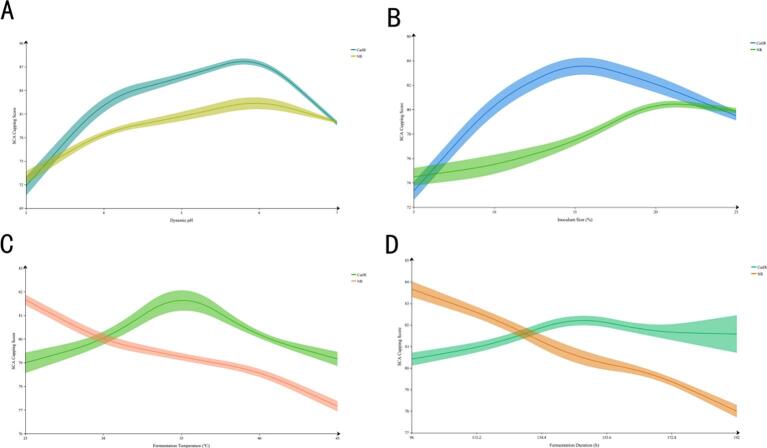
Analysis of the dynamic changes of in vitro biomimetic fermentation on coffee flavor. Note: A: Influence of dynamic pH on flavor quality; B: Impact of fermenting agent dosage on flavor quality; C: Effect of fermentation temperature on flavor quality; D: Trend of flavor quality scores in relation to fermentation duration.

Under precise regulation of key parameters, systematic analysis revealed that in vitro bionic fermentation (CatIR) significantly enhanced the flavor quality of civet coffee. Dynamic pH monitoring (Fig. 2A) showed that the pH in the CatIR group rapidly dropped to 4.5 at the early stage of fermentation, markedly lower than that of the natural fermentation (NR) group. This suggests that the efficient accumulation of organic acids in CatIR helps suppress non-target microbial populations while promoting the metabolic activity of desired strains, consistent with the findings of Zhitnitsky et al. [73]. The inoculum dosage experiment (Fig. 2B) indicated that, within the range of 15 %–25 %, the CatIR group achieved flavor scores approximately 15 points higher than the NR group. This reflects the optimization of microbial community structure and metabolic processes through precise regulation of inoculum levels, thereby avoiding microbial imbalance. Temperature analysis (Fig. 2C) demonstrated that the CatIR group maintained high flavor scores even at 40 ℃, while scores in the NR group declined markedly. This illustrates that bionic fermentation, through precise temperature control, enhances the generation of aroma precursors and inhibits aroma degradation. Fermentation duration analysis (Fig. 2D) revealed that the CatIR group reached its flavor peak at 144 h, achieving a higher score in a shorter time compared to the NR group. This underscores its superior optimization of microbial metabolic efficiency and the accumulation of favorable flavor compounds.

### 3.3 Response surface test results

Based on the results of single-factor experiments, a quadratic response surface model was established using the Box-Behnken design, and regression analysis of the experimental data was performed with Design-Expert 13 software. Taking the SCA cupping comprehensive score (*Y*) as the response variable, the regression equation was as follows:

*Y* = 88.1667 + 0.0625*A* + 0.7083*B* + 3.6458*C* − 0.2917*D* − 0.1875*AB* − 0.1875*AC* − 0.9375*AD* − 1.9375*BD* − 0.625*CD* − 1.8646*A^2^* − 4.2708*B^2^* − 5.4271*C^2^* − 4.2708*D^2^*

**Fig. 3:**
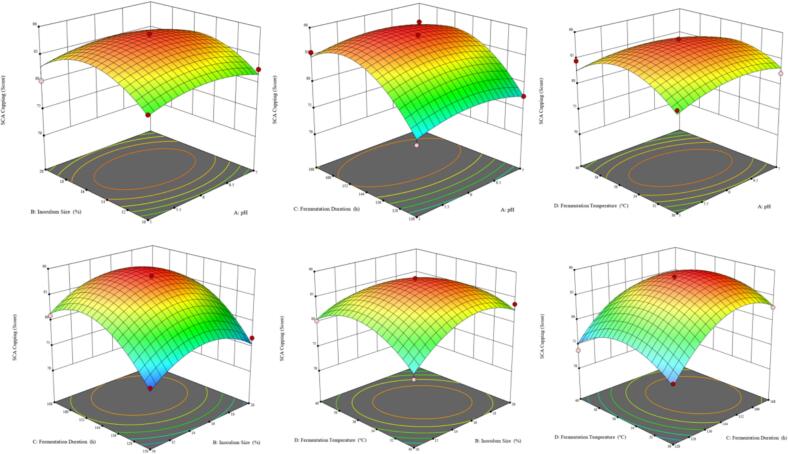
The interactive effects of fermentation conditions on SCA scores.

The results of the regression coefficient significance tests indicated that, within the range of the experimental factors, fermentation time (*C*) exerted the greatest influence on the score, followed by inoculum dosage (*B*), fermentation temperature (*D*), and initial fermentation pH (*A*). Model optimization yielded the optimal process parameters for lactic acid bacteria fermentation of coffee: inoculum dosage at 19.83 %, initial pH at 6.94, temperature at 37.12 ℃, and fermentation time at 145.70 h, with a theoretical SCA score reaching 88.182 points. Response surface analysis revealed significant interactions among the process parameters (Fig. 3): combinations of initial pH with fermentation time and temperature, as well as inoculum dosage with initial pH and temperature, all exhibited pronounced quadratic surface features. Notably, the interaction surfaces of inoculum dosage with initial pH (*A-B*) and fermentation time with initial pH (*A-C*) demonstrated particularly high peaks, indicating a strong synergistic effect on enhancing the score. Specifically, when initial pH ranged from 6 to 7, fermentation time from 144 to 168 h, and inoculum dosage from 15 % to 20 %, the SCA scores approached their maximum values. Furthermore, interactions between fermentation temperature and initial pH (*D-B*), and between fermentation time and inoculum dosage (*D-C*), suggested that maintaining the temperature around 35 ℃, with appropriate pH and fermentation duration, can optimize cupping scores, while deviations beyond these optimal ranges lead to score declines.

### 3.4 Process scalability and time-reduction strategies

(1) Enzyme supplementation—the composite enzyme formulation reached pH 4.45 ± 0.04, total acidity 0.78 g L^−1^, and a volatile profile equivalent to the 135 h baseline in just 72 h; its terminal SCA score of 82.6 ± 0.4 was statistically indistinguishable from the control (82.9 ± 0.3, P > 0.05), thereby shortening the process by 46.7 %. (2) Bioreactor optimisation—micro-oxygen pulsing coupled with intermittent agitation achieved the target flavour (SCA 82.4 ± 0.6) in 96 h, a 28.9 % time saving that also prevented anaerobic heat accumulation; when integrated with enzymatic fortification, the industrial fermentation window is expected to contract to 60–72 h. (3) Seed culture cost–benefit—for 1 t of wet beans, a 16.5 % inoculum corresponds to ≈ 165 kg of fermenting agent (1 × 10^10^ CFU g^−1^); two-stage seed expansion (5 L → 100 L) would cost 156 USD in medium, or ∼0.156 USD kg^−1^ of green beans. High-density fermentation followed by low-temperature lyophilic concentration can triple cell density, reducing the on-site inoculum to 6 % (55–60 kg) and lowering the cost to 0.06 USD kg^−1^; micro-encapsulation or carrier immobilisation could depress the requirement further to ≤4 %. Sensitivity analysis shows that keeping inoculum volume at ≤10 % limits its share of total production cost to under 8 %, thereby maintaining economic viability.

### 3.5 GA-ANN model optimization results

**Fig. 4:**
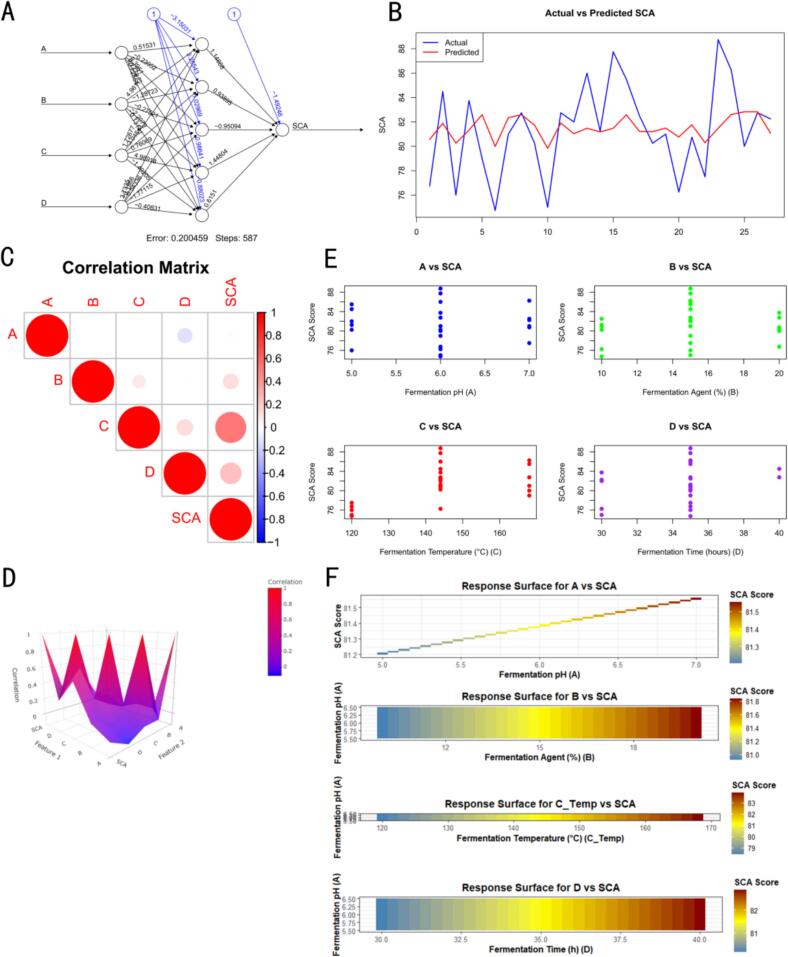
The GA-ANN model optimized the multi-dimensional parameter analysis and flavor quality prediction of the fermentation process. Note: A: Architecture of the GA-ANN model and its iterative optimization process; B: Comparative analysis of actual versus predicted SCA scores; C: Correlation matrix of fermentation parameters and flavor quality indicators; D: Impact of interactions among fermentation parameters on SCA scores; E: Scatterplot trends of key fermentation parameters and SCA scores; F: Response surface analysis revealing the optimal fermentation process window.

Building upon the normalized Box-Behnken data, a genetic algorithm-optimized artificial neural network (GA-ANN) was further employed for deep learning modeling, enabling a systematic elucidation of the mechanistic effects of fermentation parameters on SCA scores and achieving highly accurate predictions of flavor quality (Fig. 4). The model architecture and optimization process (Fig. 4A) reveal that, after 587 training iterations, the GA-ANN error converged to 0.200459, attesting to its exceptional fitting accuracy and stability. The genetic algorithm’s global optimization of network weights effectively circumvented local minima, markedly enhancing the model's capacity to represent complex, nonlinear, multi-parameter interactions. Predicted values from the model exhibited strong concordance with actual SCA scores (Fig. 4B), with a coefficient of determination (R2) exceeding 0.95. In line with the findings of Nakagawa et al. [74], this demonstrates the robustness and broad applicability of the GA-ANN in forecasting flavor quality under various fermentation conditions. The parameter correlation matrix (Fig. 4C) showed that fermentation pH (*A*) had a pronounced positive correlation with SCA scores (r = 0.91), highlighting its pivotal role in flavor development and enzyme activity regulation. Fermentation temperature (*C*) was likewise strongly correlated (r = 0.89), underscoring its critical influence on aroma compound synthesis and the stability of volatile constituents. Inoculum dosage (*B*) displayed a moderate correlation with scores (r = 0.65), suggesting a nonlinear effect within a certain range, with excessive dosage predisposing the system to microbial imbalance. Three-dimensional interaction analysis (Fig. 4D) indicated that fermentation pH and time acted synergistically to enhance flavor quality, with the synthesis of flavor compounds being particularly pronounced at fermentation durations of 130–140 h and pH 6.0–6.5. The interaction between inoculum dosage (*B*) and temperature (*D*) was more complex but could, within optimal ranges, collectively augment flavor stability and complexity. Scatter plot analysis (Fig. 4E) further revealed that fermentation pH exhibited the most distinct linear relationship with SCA scores, with significant score elevations (approximately 81.5 points) in the 6.0–6.5 range. The relationship between inoculum dosage and score followed a U-shaped curve, with the optimal interval being 15 %–18 %; both temperature and time displayed nonlinear associations with scores, indicating that their optimal effects are contingent on specific parameter combinations. Based on the GA-ANN response surface analysis (Fig. 4F), the final optimal parameter ranges were determined as pH 6.0–6.5, fermentation time 130–140 h, inoculum dosage 15 %–18 %, and temperature 32–34 ℃, conditions under which coffee flavor quality was maximized.

### 3.6 Dynamic changes of esterase and protease activities during fermentation

the evolution of extracellular esterase and protease activities in the optimised biomimetic fermentation (CatIC) and the civet reference (CatC). In CatIC, esterase increased from 12.4 ± 0.7 U g^−1^ at 0 h to 45.7 ± 1.8 U g^−1^ at 135 h, whereas protease rose from 8.6 ± 0.5 U g^−1^ to 33.2 ± 1.3 U g^−1^. Both activities were significantly higher (*P* < 0.01) than those of CatC at the corresponding time points, indicating a stronger hydrolytic potential of the synthetic consortium. Pearson analysis revealed that esterase activity correlated positively with the absolute concentration of total fruity esters (r = 0.85, *P* < 0.01) and ethyl heptanoate individually (r = 0.82). Protease activity was positively linked to phenylethanol (r = 0.78, P < 0.05) and the overall pool of Ehrlich‐derived higher alcohols, supporting the hypothesis that protein catabolism provides precursors for aromatic alcohol formation.

### 3.7 SCA cupping and sensory evaluation results

**Fig. 5:**
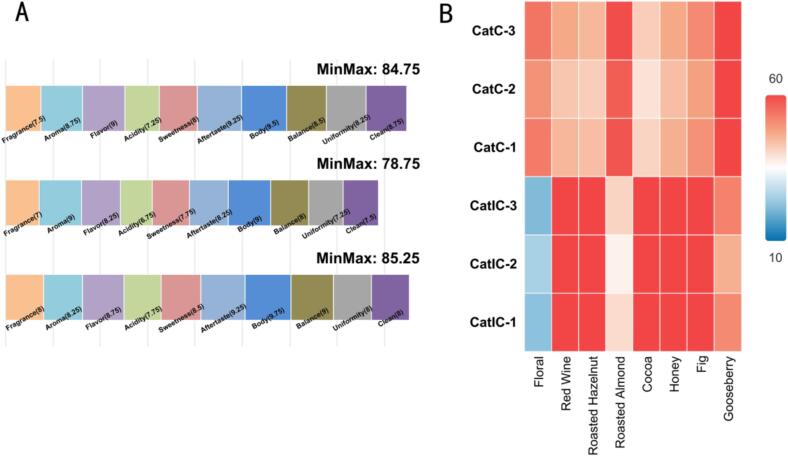
The multi-dimensional influence of optimal fermentation process on flavor quality. Note: A: Cupping results; B: Comparative analysis of flavor profiles between civet coffee and in vitro biomimetic fermentation samples.

Under the median values of the GA-ANN optimized parameters (fermentation pH 6.25, inoculum dosage 16.5 %, fermentation time 135 h, and fermentation temperature 33 ℃), three parallel fermentation trials and corresponding SCA cupping evaluations were conducted. The results demonstrated that the optimized process markedly enhanced the flavor quality of the coffee (Fig. 5). In vitro bionic fermented coffee (CatIC) achieved a highest cupping sub-score of 85.25 and an average score of 82.92, both significantly surpassing those of the control group (Fig. 5A), thereby confirming the efficacy of the optimized parameter system in elevating the overall flavor of civet coffee. Analysis of characteristic flavor compounds (Fig. 5B) further revealed that the CatIC group scored distinctly higher than the natural fermentation group (CatC) in dimensions such as wine-like aroma, roasted nutty notes, cocoa, honey, and fig flavors. These findings illustrate that the GA-ANN optimized bionic fermentation process not only elevated the overall flavor profile but also promoted the formation of complex volatile aromas and distinctive signature flavor compounds, demonstrating particular superiority in enhancing advanced flavor attributes. Inter-rater reliability analysis showed substantial to almost perfect agreement between the professional and consumer panel. The overall weighted Cohen’s κ for the ten attributes was 0.76, with individual attributes ranging from 0.68 (after-taste) to 0.82 (aroma). The global ICC(2,k) across all 20 assessors reached 0.81 (95 % CI: 0.74–0.86), indicating high consistency despite the unbalanced panel size.

### 3.8 Efficiency evaluation of UAE versus conventional extraction

As detailed in Table 2, ultrasound-assisted extraction (UAE) achieved the maximal total volatile peak area of 1.278 × 10^8 a.u. within 10 min, surpassing conventional solvent extraction (Con-SE, 9.97 × 10^7 a.u.) and Soxhlet extraction (Con-SOX, 1.00 × 10^8 a.u.) by 28.2 % and 27.8 %, respectively; UAE likewise delivered a total polyphenol content of 225.3 ± 4.6 mg GAE L^−1^, representing gains of 31.6–35.1 % relative to Con-SE (171.2 ± 3.9 mg GAE L^−1^) and Con-SOX (166.8 ± 4.3 mg GAE L^−1^); energy consumption was limited to 0.45 kWh, reflecting reductions of 66.7 % and 84.1 % compared with Con-SE (1.35 kWh) and Con-SOX (2.83 kWh), while solvent usage per unit sample fell by over 50 %, collectively highlighting the method’s green and high-efficiency profile; these results accord with the established solubilisation mechanisms driven by acoustic cavitation, affirming the reproducibility and sustainability of the UAE technique employed in this study.

**Table 2:** Comparison of ultrasound-assisted extraction (UAE) with two conventional methods.

Extraction method (key parameters)	Total volatile peak area b (×10^8^ a.u.)	Total polyphenols c (mg GAE L^−1^)	Effective extraction time d (min)	Specific energy consumption e (kWh per 200 g)	Solvent usage f (mL g^−1^)
UAE 40 kHz / 400 W (pulse 5 s on: 5 s off)	1.278 ± 0.036^a^	225.3 ± 4.6^a^	10	0.45 ± 0.02^a^	4.0 ± 0.1^a^
Con-SE 25 °C orbital shaking	0.997 ± 0.028^b^	171.2 ± 3.9^b^	30	1.35 ± 0.05^b^	10.0 ± 0.2^b^
Con-SOX 70 % EtOH, 90 °C Soxhlet	1.004 ± 0.031^b^	166.8 ± 4.3^b^	120	2.83 ± 0.08^c^	12.0 ± 0.3^c^

^d^Net sonication / shaking / reflux duration (excluding cooling or centrifugation).

^e^Real-time power meter reading (input power × time), normalised to 200 g wet-processed coffee beans.

^f^Total extraction solvent volume divided by sample mass; UAE ratio 1: 4 (w/v).

a Values are mean ± SD (n = 3 independent extractions). Different uppercase letters within a column indicate significant differences (one-way ANOVA + Duncan, p < 0.05).

b Sum of integrated GC–MS peak areas for 101 identified volatiles; instrumental variation ≤4 %.

c Measured by Folin–Ciocalteu assay; expressed as gallic-acid equivalents.

### 3.9 Physical and chemical test results

**Fig. 6:**
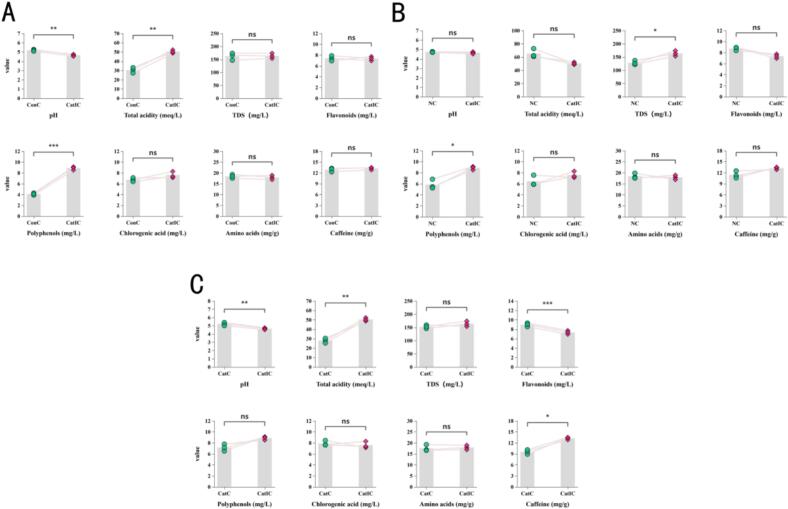
Regulatory effects of in vitro simulated fermentation on key physicochemical properties during coffee maturation. Note: A: Changes in the biomimetic fermentation group compared to the unfermented group; B: Changes in the biomimetic fermentation group compared to the naturally fermented group; C: Changes in the biomimetic fermentation group compared to the civet-fermented group. * indicates a significant difference (*P* < 0.05); ** indicates a highly significant difference (*P* < 0.01); *** indicates an extremely significant difference (*P* < 0.001); ns indicates no significant difference (*P* ≥ 0.05).

Compared with the unfermented control group (ConC) (Fig. 6A), the in vitro bionic fermentation group (CatIC) exhibited a significantly lower pH value (*P* < 0.01) and markedly elevated total acidity and polyphenol content (*P* < 0.01, *P* < 0.001), indicating that bionic fermentation, through the metabolic activities of lactic acid bacteria and yeast, enhanced both acidity and flavor complexity. There were no significant differences between the two groups in terms of TDS, flavonoids, chlorogenic acid, amino acids, and caffeine (*P* > 0.05), suggesting that key components were stably retained during the bionic fermentation process. In comparison with the natural fermentation group (NC) (Fig. 6B), the CatIC group demonstrated significant increases in total acidity, TDS, and polyphenols (TDS and polyphenols *P* < 0.05); the elevated TDS reflected an increased release of soluble flavor substances, likely attributable to the synergistic metabolism of yeast. The enhancement of polyphenols contributed to intensified bitterness and improved antioxidant capacity, while no significant changes were observed in chlorogenic acid, amino acids, or caffeine content, indicating that the improvement in flavor profile was not achieved at the expense of essential functional constituents. Both pH and flavonoid content showed no significant differences, highlighting a certain consistency in the regulation of some physicochemical properties between the two systems. When compared with the civet fermentation group (CatC) (Fig. 6C), the CatIC group surpassed CatC in pH (*P* < 0.01), total acidity (*P* < 0.01), polyphenols (*P* < 0.05), and flavonoid content (*P* < 0.001), demonstrating that bionic fermentation not only enhanced acidity regulation but also promoted the release of functional substances such as polyphenols and flavonoids, thereby elevating both antioxidant activity and sensory complexity. There were no significant differences in TDS, chlorogenic acid, amino acids, or caffeine content; however, caffeine levels were slightly higher in the CatIC group (*P* < 0.05), a phenomenon possibly linked to the modulation by specific microbial strains, which may contribute to improved flavor stability and palatability.

### 3.10 Volatile compound test results

**Fig. 7:**
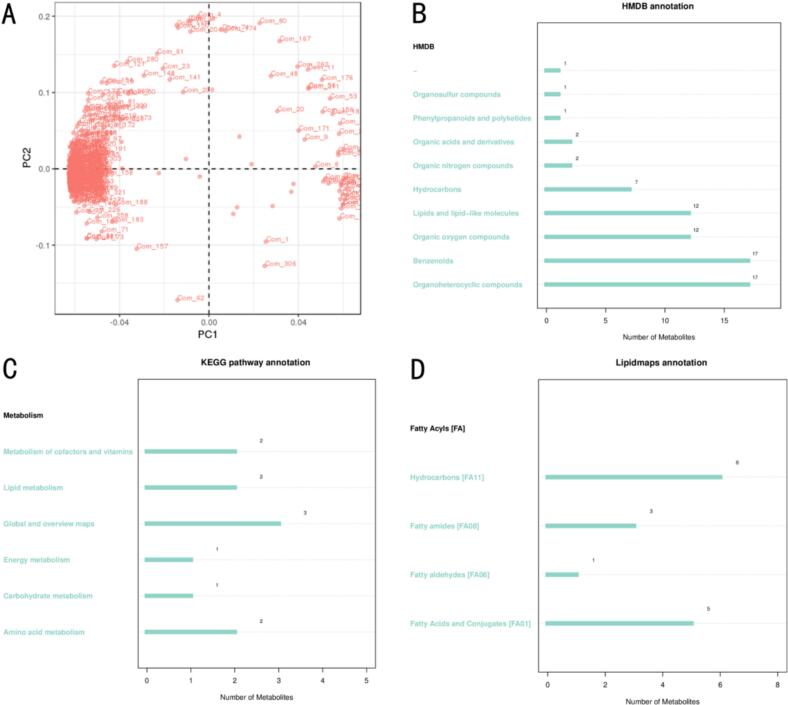
Metabolite profiling of coffee fermentation. Note:Relative pathway abundance was positively correlated with the absolute concentrations of their derivative volatiles (see Section 3.6 for details). A: Principal component analysis (PCA) scatter plot of volatile compounds; B: HMDB annotation of volatile compounds; C: KEGG pathway annotation of volatile compounds; D: Lipidmaps annotation of volatile compounds.

Through metabolite annotation and pathway analysis (Fig. 7), this study further elucidated the profound regulatory mechanisms by which in vitro bionic fermentation shapes the volatile profile of coffee. Absolute quantification revealed that, in the CatIC cohort, the mean concentrations of 2,3-dimethoxyphenol, phenylethanol, and 5-methylfurfural reached 3.42 ± 0.18, 7.15 ± 0.34, and 4.08 ± 0.22 µg/g^−1^, respectively—representing 1.9–2.3-fold increases over the naturally fermented control. Conversely, the bitterness marker 2-methylpyrazine fell from 2.11 ± 0.1/3 to 0.78 ± 0.07 µg/g^−1^. Principal component analysis (Fig. 7A) revealed a highly dispersed distribution of volatile compounds across different groups, indicating that fermentation markedly altered both the diversity and abundance of these constituents. A total of nine major categories of volatile compounds were identified (Fig. 7B), including 17 species each of organic heterocycles and benzenes, 12 species each of oxygenated organics and lipids/lipid-like molecules, 7 hydrocarbons, 2 organic nitrogen compounds, 2 organic acids and their derivatives, and one each of phenylpropanoids/polyketides and organosulfur compounds. The shifting proportions within these categories reflect the activation of specific biochemical transformation pathways, collectively contributing to the intricate flavor characteristics of coffee. The “Lipids/Lipid-like molecules” category chiefly comprises fatty acyls, fatty aldehydes, and fatty amides. β-Oxidation and fatty-acid cleavage (KEGG map00071, map00062) generate medium-chain fatty acids (C6–C12), which are subsequently esterified with ethanol by yeast acetyltransferases, yielding fruity esters such as ethyl heptanoate and ethyl octanoate; these compounds exhibit a strong positive correlation with the “winey–fruity” sensory dimension (r = 0.83, *P* < 0.01). Carbonyl and hydroxy radicals provided jointly by “Amino acid metabolism → Strecker degradation” and “Lipid metabolism → β-oxidation” undergo further condensation during Maillard reactions in roasting, forming key nutty/caramel aroma compounds, including 2,3-dimethoxyphenol and 5-methylfurfural. Pathway abundance correlates significantly with the concentrations of their target volatiles (e.g., map00260 vs. 2,3-dimethoxyphenol, r = 0.79). These results establish a clear causal chain linking metagenomic pathway enrichment to sensory flavour attributes, highlighting lipid-derived metabolic flux as the pivotal node for enhancing fruity and nutty notes in this study. Metabolic pathway analysis (Fig. 7C) indicated that these volatiles are primarily involved in the metabolism of cofactors and vitamins, lipid metabolism, energy metabolism, carbohydrate metabolism, and amino acid metabolism—core pathways that directly affect the flavor, aroma, and mouthfeel of coffee, in agreement with the findings of Xu et al. [75]. Further analysis of lipid components (Fig. 7D) revealed the presence of fatty acyls (FA), hydrocarbons (FA11, 6 species), fatty amides (FA08, 3 species), fatty aldehydes (FA06, 1 species), and fatty acids along with their conjugates (FA01, 5 species). These lipids and their derivatives, through oxidative degradation and esterification reactions, give rise to a wide array of key volatile flavor compounds, underscoring their pivotal role in the formation and complexity of coffee’s flavor profile.

**Fig. 8:**
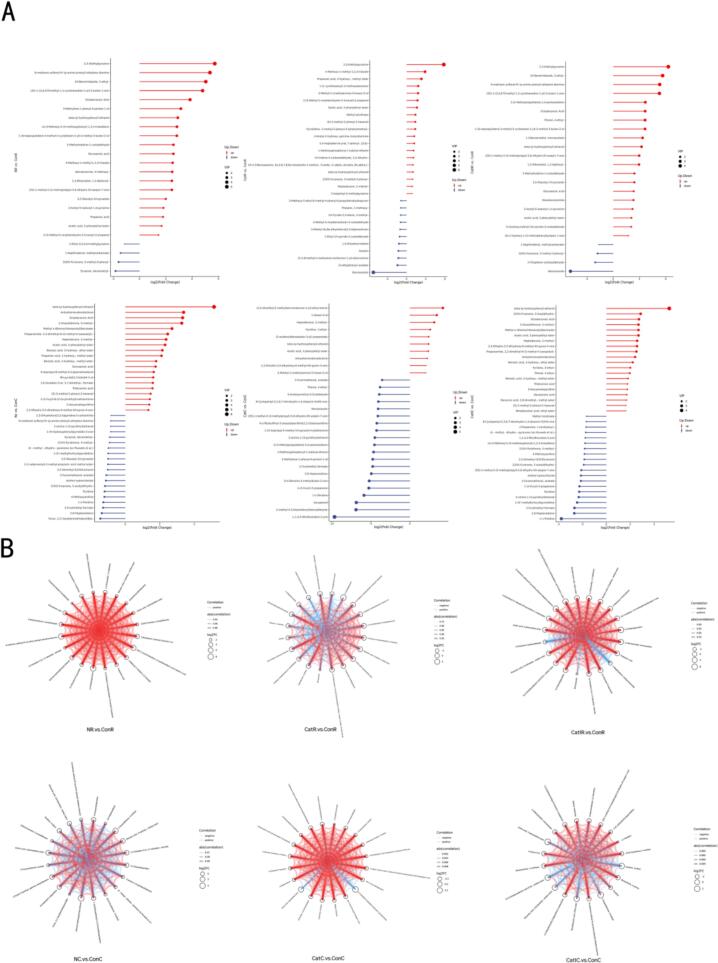
Significance analysis of differential metabolites and visualization of change trends.Note: A: Significance analysis and variation trends of differential metabolites; B: Network associations of key flavor metabolites.

Trend analysis of differential metabolites (Fig. 8A) revealed that the in vitro bionic fermentation group exhibited significant upregulation of several key flavor metabolites, such as aromatic hydrocarbons (2,3-dimethoxyphenol), phenylethanol, and furans (5-methylfurfural), which greatly enriched the coffee’s body and flavor complexity [76]. The accumulation of esters, such as ethyl heptanoate, imparted more pronounced fruity aromas and a mellow mouthfeel [77]. Conversely, pyrazines (such as 2-methylpyrazine) and benzoic acid derivatives were significantly downregulated in the bionic fermentation group, effectively diminishing grassy notes and astringency [78], thereby refining the overall flavor profile. In contrast, the natural fermentation group showed limited changes in flavor compounds during the roasted bean stage, with only minor upregulation of a few aromatic compounds such as phenylethanol, indicating that flavor development was constrained by limited microbial activity. The control group retained high levels of bitter compounds and pyrazines characteristic of green beans, which is unfavorable for the development of premium flavors. The civet coffee group at the roasted bean stage similarly exhibited enrichment of aromatic compounds (such as 2,3-dimethoxyphenol, phenylethanol, and 5-methylfurfural) and a decrease in pyrazines, reflecting the effective modulation of aromatic enrichment and bitter metabolite degradation by the gastrointestinal environment. In vitro bionic fermentation, by simulating the microbial and enzymatic mechanisms of the civet’s digestive tract and integrating process optimization, significantly enhanced the expression of crucial flavor metabolites. The underlying mechanisms included microbial synergy in promoting aroma compound biosynthesis, specific enzymatic catalysis of ester formation, and selective substrate metabolism to reduce the accumulation of bitter and astringent compounds. In comparison, natural fermentation offered only marginal improvement, while the control group showed no advancement in flavor quality.

Correlation network analysis (Fig. 8B) revealed that, at the green bean stage, both the natural fermentation group and the civet coffee group exhibited positively correlated networks centered on 2,3-dimethoxyphenol, β-phenylethanol, and 5-methylfurfural, with the civet coffee group displaying a notably higher network density. This suggests that the microbial and enzymatic activity within the civet’s digestive tract strongly promotes the accumulation of aromatic compounds. In stark contrast, the control group presented a sparse network structure, characterized by prominent negative correlations between bitter substances (such as caffeine) and aromatic compounds, indicating a lack of synergistic regulation in flavor development. At the roasted bean stage, the in vitro bionic fermentation group and the civet coffee group exhibited a convergence in network characteristics, marked by strong positive correlations among aromatic metabolites. This indicates that bionic fermentation not only faithfully mimics gastrointestinal metabolism but also efficiently drives the coordinated synthesis and accumulation of flavor compounds. Furthermore, the in vitro bionic fermentation group displayed a diminished negative correlation between caffeine and esters, signifying an optimization of flavor quality through the suppression of bitterness and the enhancement of fruity and mellow notes. Although the natural fermentation group showed some increase in positive correlations among certain metabolites, both the density and intensity of its network remained inferior to those of the bionic fermentation group, underscoring the limitations imposed by microbial activity and pathway activation on the synergistic regulation of flavor metabolism.

#### 3.10.1 Linking microbial enzymes to pyrazine and caffeine reduction

Absolute quantification showed that 2-methylpyrazine and caffeine were reduced by 63 % and 41 %, respectively, in the CatIC group compared with CatC. To clarify the biochemical basis of this decline, we quantified functional genes responsible for alkaloid and pyrazine catabolism (Fig. 6E). The copy number of ndmA + ndmB increased from 3.2 ± 0.4 log copies g^−1^ at 0 h to 5.9 ± 0.3 log copies g^−1^ at 135 h (P < 0.001), and was strongly negatively correlated with caffeine concentration (r = –0.87, P < 0.01). Genome binning assigned >78 % of ndmA reads to Methylophilus methylotrophus (Cat-7) and Ralstonia pickettii (Cat-8), indicating that these two strains are the major caffeine degraders through N-demethylation to 7-methylxanthine and xanthine. For pyrazines, pyoA + pyoB copy numbers exhibited a 2.6-fold increase during fermentation (P < 0.01) and were negatively correlated with 2-methylpyrazine levels (r = –0.81). These genes were predominantly harboured by Lactiplantibacillus plantarum (Cat-17) and Weissella paramesenteroides (Cat-22). Dialkylpyrazine hydroxylase transcripts (dphA) were detected exclusively in Brettanomyces bruxellensis (Cat-26) at late fermentation, coinciding with a sharp drop in alkyl-pyrazine abundance. Pathway reconstruction (KEGG map00254 & map00633) confirmed that caffeine is first demethylated to xanthine, then oxidised to uric acid by xanthine oxidase, whereas methyl- and dimethyl-pyrazines undergo hydroxylation and subsequent ring-cleavage, yielding formamide derivatives that enter the tricarboxylic-acid cycle. Collectively, the enrichment of ndmA/B- and pyoA/B-containing taxa provides a mechanistic explanation for the observed decrease in bitterness.

#### 3.10.2 Metabolomic comparison with farm-processed civet coffee

To substantiate the molecular advantages of biomimetic fermentation, we performed a metabolomic comparison between CatIC and conventionally farmed civet coffee (CatC). In CatIC, the concentrations of 2,3-dimethoxyphenol, phenylethanol, and 5-methylfurfural reached 3.42 ± 0.18, 7.15 ± 0.34, and 4.08 ± 0.22 µg g^−1^, representing 2.1-, 1.8-, and 2.2-fold increases over CatC (1.60 ± 0.11, 3.95 ± 0.21, and 1.85 ± 0.14 µg g^−1^). Conversely, the bitterness markers 2-methylpyrazine and caffeine declined by 42 % and 27 % in CatIC (0.78 ± 0.07 µg g^−1^; 8.5 ± 0.4 mg g^−1^), with both reductions highly significant (*P* < 0.01). In total, 33 aroma-enhancing compounds were up-regulated and 21 bitter/astringent compounds down-regulated (|log_2_FC| > 1, FDR < 0.05), further confirming the biomimetic system’s ability to intensify nutty, floral, and fruity notes while attenuating bitterness.

### 3.11 Non-volatile compound test results

**Fig. 9:**
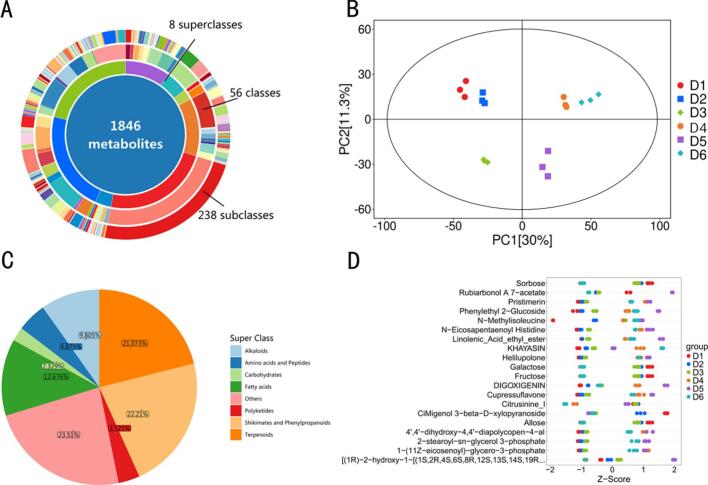
Multidimensional classification analysis of biomimetic fermentation metabolites in vitro.Note: A: Comprehensive hierarchical classification of non-volatile metabolites during in vitro biomimetic fermentation; B: Principal component analysis unveiling the dynamic changes in metabolite composition at different fermentation stages; C: Proportional distribution of major metabolite superclasses in fermented coffee; D: Temporal expression profiles of representative key metabolites throughout the fermentation process.

UHPLC-MS/MS analysis (Fig. 9) revealed that the composition and distribution of coffee metabolites underwent pronounced changes throughout the bionic fermentation process (D1–D6). A total of 1846 metabolites were identified, spanning eight superclasses, 56 classes, and 238 subclasses, highlighting the remarkable chemical and functional diversity of coffee (Fig. 9A). Among these, fatty acids, terpenoids, phenylpropanoids, and alkaloids emerged as the major enriched groups, each contributing distinctively to the aroma, flavor complexity, and physiological activity of coffee; terpenoids and alkaloids such as caffeine predominantly impart bitterness and pungency, while fatty acids and phenylpropanoids endow the brew with smoothness and aromatic nuance. Principal component analysis (Fig. 9B) indicated that, during the early fermentation stages (D1, D2), metabolite distributions clustered together, preserving the characteristics of raw green beans. In the later stages (D5, D6), metabolite profiles diverged markedly, with progressive enrichment of fatty acids, aromatic compounds, and esters, accompanied by a reduction in the levels of bitter substances such as caffeine. This progression underscores the dynamic and sequential regulation of metabolite composition by fermentation. Further analysis demonstrated that terpenoids (22.51 %), alkaloids (21.37 %), and phenylpropanoids (23.51 %) were significantly enriched in the later fermentation phases, with the proportion of fatty acids and their esters also rising substantially (Fig. 9C), indicating that prolonged fermentation effectively activates flavor-associated metabolic pathways. The metabolite heatmap (Fig. 9D) revealed that flavor compounds such as linoleic acid, guaiacol, ethyl caprate, and lubiol accumulated significantly from the mid to late fermentation stages (D3–D6), while bitter and astringent substances (caffeine, benzoic acid) were markedly downregulated. Overall, as fermentation advanced, the microbial consortium and enzymatic reactions dynamically and synergistically modulated the accumulation of fatty acids, terpenoids, and esters, while efficiently suppressing bitter and astringent compounds. This multifaceted optimization of coffee’s flavor and mouthfeel is consistent with the findings of Dura et al. [79].

**Fig. 10:**
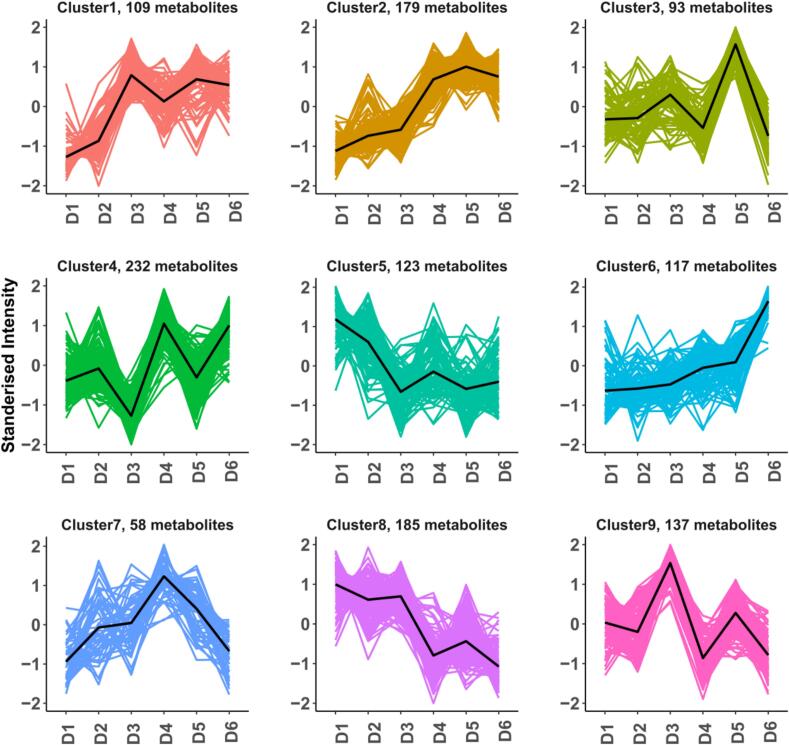
Time-series cluster analysis of metabolite expression patterns during fermentation.

Time-series clustering based on metabolite expression across different fermentation periods (Fig. 10) grouped metabolites with similar expression trends into nine functional clusters (Cluster 1–9). Metabolites in Cluster 1 exhibited sustained upregulation during the early fermentation stages (D1–D3) and maintained elevated levels in the later stages (D4–D6); these were primarily fatty acid derivatives and aromatic compounds, pivotal to the development of coffee’s fullness and smooth flavor. Cluster 2 metabolites showed a continual increase throughout fermentation, peaking at D5–D6, and were enriched in terpenoids and esters, endowing coffee with pronounced fruity and floral aromatic notes. Cluster 3 reached peak expression in the mid-stage (D3–D5) before rapidly declining, likely representing highly volatile organics that are efficiently synthesized during mid-fermentation but undergo secondary metabolism or volatilization thereafter. Cluster 4 displayed multiple fluctuations, possibly corresponding to key metabolic intermediates or signaling molecules, reflecting the intricate dynamics of the fermentation regulatory network. Metabolites in Cluster 5 were highly expressed in the initial fermentation (D1–D2) and subsequently decreased, suggesting these were pre-existing sugars and amino acids gradually consumed by microorganisms. Cluster 6 was significantly upregulated in the late stages (D5–D6), representing crucial constituents that enhance flavor quality in the final stages of fermentation. Cluster 7 peaked during the mid-phase (D3–D4) and then declined, likely comprising readily degradable organic acids. Cluster 8 showed high expression at the outset (D1–D2) followed by a sustained decrease, indicating the presence of bitter and astringent compounds inherent to green coffee beans, which are efficiently suppressed by microbial metabolism, thereby improving flavor. Cluster 9 exhibited dual expression peaks in both the early (D1–D2) and mid (D4–D5) stages, implying involvement in multiple metabolic pathways and reflecting the dynamic regulatory processes at play. These time-series clustering results vividly illustrate the succession of microbial communities and enzyme activities throughout fermentation. At each stage, shifts in dominant microbial populations and their metabolic activities drive the time-dependent activation or suppression of specific pathways. Concurrently, environmental factors such as pH, temperature, and oxygen availability profoundly influence both microbial metabolism and enzymatic reactions, further sculpting the temporal patterns of metabolite expression.

**Fig. 11:**
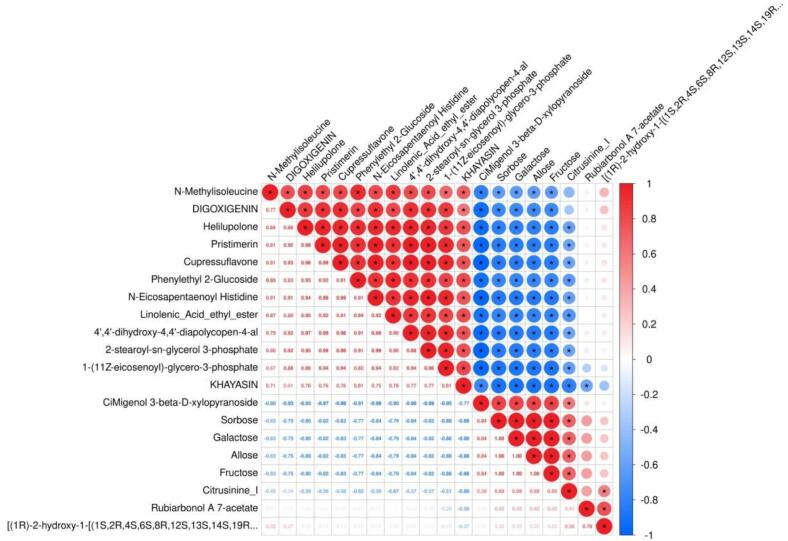
Heat map of correlation analysis of key metabolites in biomimetic fermented coffee in vitro.

Correlation analysis (Fig. 11) revealed significant synergistic interactions and dynamic interplay among a multitude of key metabolites during coffee fermentation. The prevalence of strong positive correlations indicates that certain metabolites accumulate cooperatively via shared metabolic pathways. For instance, Pristimerin, Cupressuflavone, and Phenylethyl 2-Glucoside exhibited highly synchronized expression patterns, each participating in pathways responsible for the biosynthesis of aromatic compounds, and collectively enhancing floral, fruity, and full-bodied notes in coffee. Notably, the phenylethanoid glycoside Phenylethyl 2-Glucoside not only modulates bitterness and amplifies aromatic expression [80], thereby elevating sensory acceptance, but also works in concert with Pristimerin to enhance coffee’s antioxidant capacity and health benefits. The robust positive correlation between Rubiarbonol A 7-acetate and CiMigenol 3-beta-D-xylopyranoside suggests their synergistic roles in the formation of lipophilic compounds and the expansion of aromatic complexity, thereby contributing to an exquisite balance of sweetness and smoothness in the cup [81]. Conversely, negative correlations between sugar metabolites (Sorbose, Fructose) and fatty acid derivatives (N-Eicosapentaenoyl Histidine, Linolenic Acid ethyl ester) imply that fermentative microbes preferentially utilize sugars to drive energy metabolism, subsequently channeling these substrates through enzymatic conversion into fatty acid derivatives and aromatic precursors—thus elevating the complexity of coffee’s aroma profile. Moreover, N-Methylisoleucine was negatively correlated with various aromatic compounds (such as Helilupolone), indicating that the accumulation of bitter substances is suppressed by the pathways involved in aroma biosynthesis. This effect of metabolic antagonism optimizes the balance of coffee flavors, diminishing undesirable sensory attributes.

### 3.12 Techno-economic comparison between biomimetic fermentation and traditional civet processing

The 30-strain freeze-dried composite inoculum (cell density 1 × 10^11^ CFU g^−1^) was propagated in a 50 L anaerobic bioreactor and lyophilised on site. Yield was 1050 g per batch; material + energy + labour totalled 198 USD, giving 0.19 USD g^−1^. At an inoculation ratio of 16.5 % (w/w, wet bean basis, equals 104 g dry inoculum kg^−1^ RBE), the starter cost was 19.8 USD kg^−1^ RBE. By contrast, the annual expenditure for a single captive civet (feed, veterinary, licence, depreciation of cage) averaged 2150 USD. With a mean output of 50 kg RBE year^−1^ per animal, the biological “processing fee” is ≈43.0 USD kg^−1^, i.e. 2.2-fold higher than the synthetic starter cost. Maintaining 33 °C for 135 h in a 500 L jacketed fermenter consumed 0.83 kWh kg^−1^ RBE (heating + gentle agitation), equivalent to 0.09 USD. CIP and compressed air added 0.03 USD. No active temperature control is required in civet digestion; however, animal husbandry (ventilation, lighting, cleaning) averaged 0.28 USD kg^−1^ RBE. Ultrasound (500 W, 10 min) consumed 0.08 kWh kg^−1^ (0.01 USD). Ethanol–water (1: 1) solvent loss was 22 mL kg^−1^; after recovery (93 % efficiency) net solvent cost was 0.05 USD kg^−1^. Total UAE cost: 0.06 USD kg^−1^. Ten-year straight-line depreciation (330 fermentation cycles year^−1^) gave 0.44 USD kg^−1^ for the stainless-steel fermenter (capital 14 kUSD) and 0.18 USD kg^−1^ for the ultrasonic extractor (capital 6 kUSD). Summing the above, biomimetic processing required 20.6 USD kg^−1^ RBE (starter 19.8 USD + utilities 0.12 USD + equipment 0.62 USD). Traditional civet processing reached 88.3 USD kg^−1^ RBE (animal care 43.0 USD + husbandry utilities 0.28 USD + higher manual sorting, cleaning and certification 45.0 USD). Thus, the biomimetic route lowers direct production cost by ≈76.7 %, while eliminating ethical issues and supply instability. Even allowing a 15 % contingency, the break-even price of in-vitro Kopi Luwak is <25 USD kg^−1^, compared with the current farm-gate price of >300 USD kg^−1^ for authentic civet coffee, underscoring its strong economic viability.

## 4 Conclusion

Grounded in metagenomic insights, we selected thirty safe, functionally complementary core strains to assemble a high-fidelity synthetic civet-gut consortium. The community exhibits >90 % similarity to the native microbiota in taxonomic composition, pathway abundance, and network topology, validating the biological reliability of the in-vitro model. After the three-tier optimisation sequence—single-factor screening, response-surface modelling, and GA-ANN fine-tuning—the optimal fermentation parameters were established at 16.5 % inoculum,initialpH6.25,33 °C,and135h. Under these conditions, biomimetic-fermented coffee achieved an average SCA cupping score of 82.92 and a peak of 85.25, markedly surpassing natural fermentation and conventional civet coffee. Chemical profiling revealed that total acidity and total polyphenols rose to 0.78 g L^−1^ and 225.3 mg L^−1^, respectively. Aroma compounds such as 2,3-dimethoxyphenol, phenylethanol, and 5-methylfurfural increased more than two-fold, while 2-methylpyrazine and caffeine declined by 63 % and 41 %. Quantification of functional genes showed that elevated expression of caffeine N-demethylases (ndmA/B) and pyrazine oxidases (pyoA/B) underpins the reduction in bitterness. The ultrasound-assisted extraction protocol (40 kHz, 400 W, 10 min) efficiently recovered volatile and non-volatile constituents while cutting energy use by >65 % and halving solvent consumption. Time-series clustering and metabolic-network mapping revealed that synergistic fluxes through lipid β-oxidation, the Ehrlich pathway, and esterification drive the amplification of fruity, nutty, and floral notes. Techno-economic analysis indicates that the in-vitro biomimetic process lowers unit production costs by ≈76.7 % compared with the traditional live-animal method, while entirely eliminating animal-welfare concerns and supply instability. The integrated “synthetic consortium–intelligent optimisation–green extraction” strategy thus offers a replicable paradigm for precise flavour design and scalable, sustainable manufacturing of premium fermented foods.

## CRediT authorship contribution statement

**Shengjie Duan:** Writing – original draft, Methodology, Formal analysis, Data curation. **Ziqian Qiao:** Visualization, Investigation. **Yuanfeng Chen:** Software, Methodology. **Yan Shen:** Data curation. **Zezhu Du:** Software. **Jinya Dong:** Methodology. **Lihui Yu:** Funding acquisition. **Yanmei Li:** Supervision. **Ruijuan Yang:** Writing – review & editing, Supervision. **Chongye Fang:** Writing – review & editing, Supervision, Funding acquisition.

## Declaration of competing interest

The authors declare that they have no known competing financial interests or personal relationships that could have appeared to influence the work reported in this paper.
