# Supplementary material for: Ultrasound-assisted extraction and flavor quality assessment of in vitro biomimetically fermented Kopi Luwak
Source: Ultrason Sonochem. 2025 Aug 6;120:107499. doi: 10.1016/j.ultsonch.2025.107499 (PMC12357160; doi:10.1016/j.ultsonch.2025.107499)

**Suppl. S1** Diagram of the finished product of the starter culture of the direct injection compound culture

The direct-inoculation compound fermentation agent, prepared using freeze-drying technology, is presented in the form of a “compound microbial freeze-dried powder.” This preparation contains 30 strains of dominant functional microorganisms, including fungi, bacteria, and molds, with 10% skimmed milk powder serving as the primary protective agent. The quality indicators at the time of release are as follows: total viable cell count ≥ 1.0 × 10¹¹ CFU·g⁻¹, of which lactic acid bacteria ≥ 8.0 × 10¹⁰ CFU·g⁻¹, yeast ≥ 1.0 × 10⁹ CFU·g⁻¹, mold spores ≥ 1.0 × 10⁸ CFU·g⁻¹; freeze-drying survival rate ≥ 85%, and moisture content ≤ 5%.

Left: Fermentation agent dry powder; right: Compound microbial suspension.


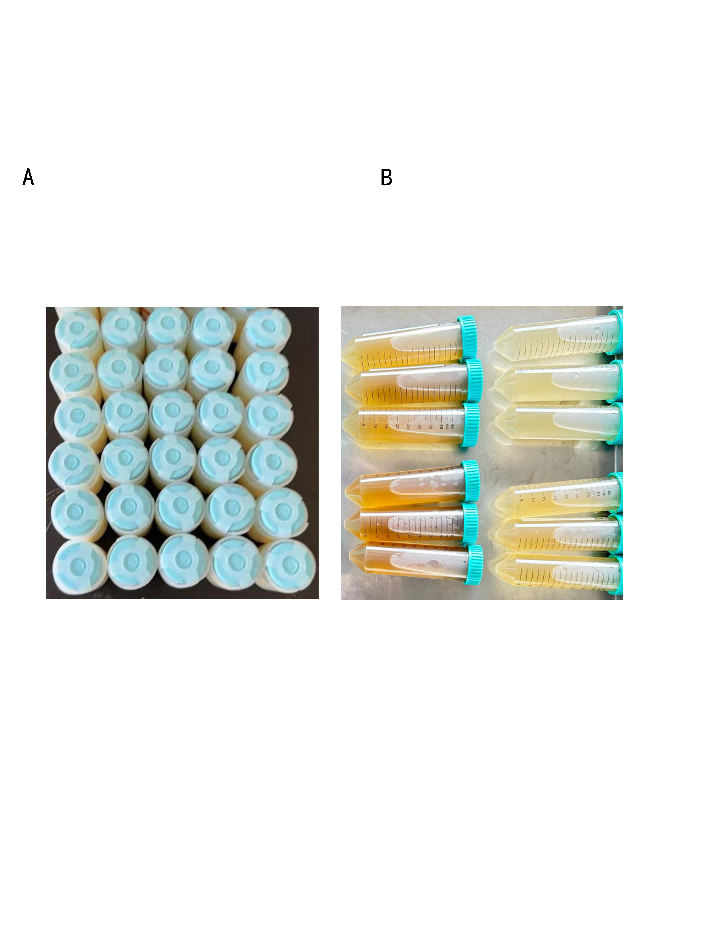

Supplement: Supplementary Data 1 [file mmc1.docx]
