# Supplementary material for: Ultrasound-assisted extraction and flavor quality assessment of in vitro biomimetically fermented Kopi Luwak
Source: Ultrason Sonochem. 2025 Aug 6;120:107499. doi: 10.1016/j.ultsonch.2025.107499 (PMC12357160; doi:10.1016/j.ultsonch.2025.107499)
Supplement: Supplementary Data 2 [file mmc2.docx]

**Suppl. S2** Analysis of variance of response surface regression model

| Source of variance | Sum of squares | Degrees of freedom | Mean square | *F*-value | *P*-value | Significance |
| --- | --- | --- | --- | --- | --- | --- |
| Model | 407.99 | 14 | 29.14 | 9.34 | 0.0002 | ** |
| *A* | 0.05 | 1 | 0.05 | 0.02 | 0.9045 |  |
| *B* | 6.02 | 1 | 6.02 | 1.93 | 0.1900 |  |
| *C* | 159.51 | 1 | 159.51 | 51.13 | <0.0001 | ** |
| *D* | 1.02 | 1 | 1.02 | 0.33 | 0.5778 |  |
| *AB* | 0.14 | 1 | 0.14 | 0.05 | 0.8354 |  |
| *AC* | 0.14 | 1 | 0.14 | 0.05 | 0.8354 |  |
| *AD* | 3.52 | 1 | 3.52 | 1.13 | 0.3093 |  |
| *BC* | 0.00 | 1 | 0.00 | 0.00 | 1.0000 |  |
| *BD* | 15.02 | 1 | 15.02 | 4.81 | 0.0487 | * |
| *CD* | 1.56 | 1 | 1.56 | 0.50 | 0.4926 |  |
| *A^2^* | 18.54 | 1 | 18.54 | 5.94 | 0.0313 | * |
| *B^2^* | 97.28 | 1 | 97.28 | 31.19 | <0.0001 | ** |
| *C^2^* | 157.08 | 1 | 157.08 | 50.36 | <0.0001 | ** |
| *D^2^* | 97.28 | 1 | 97.28 | 31.19 | <0.0001 | ** |
| Residual | 37.43 | 12 | 3.12 |  |  |  |
| Lack of fit | 36.89 | 10 | 3.69 | 13.62 | 0.0703 |  |
| Absolute error | 0.54 | 2 | 0.27 |  |  |  |
| Total | 445.42 | 26 |  |  | *CV* = 2.18% | |
