# Supplementary material for: Ultrasound-assisted extraction and flavor quality assessment of in vitro biomimetically fermented Kopi Luwak
Source: Ultrason Sonochem. 2025 Aug 6;120:107499. doi: 10.1016/j.ultsonch.2025.107499 (PMC12357160; doi:10.1016/j.ultsonch.2025.107499)
Supplement: Supplementary Data 3 [file mmc3.docx]

**Suppl. S3** Examination of Calibration Curves and Corresponding Formulas.

| Index | Standard curve | Calculation formula | Notation |
| --- | --- | --- | --- |
| Flavonoids（mg/L） | y=0.0238x-0.0238 | *C*=0.0238·*A*−0.0238 | *C*：Flavonoid concentration（mg/L）；  *A*：absorbance |
| Polyphenols（mg/L） | y=−0.0548x+1.5 | *C*=−0.0548·*A*+1.5 | *C*：polyphenol concentration（mg/L）；  *A*：absorbance |
| Chlorogenic acid（mg/L） | y=0.0235x+0.0123 | *C*=0.0235·*A*+0.0123 | *C*：Chlorogenic acid concentration（mg/L）；  *A*：absorbance |
| Amino acids（mg/g） | y=0.0152x+0.0104 | *C*=0.0152·*A*+0.0104 | *C*：Amino acid concentration（mg/g）；  *A*：absorbance |
| Caffeine (mg/g) | y=0.4142x+0.0000 | *C*=0.4142·*X* | *C*：Caffeine concentration（mg/g）；  *X*：Peak area |
