# Supplementary material for: Ultrasound-assisted extraction and flavor quality assessment of in vitro biomimetically fermented Kopi Luwak
Source: Ultrason Sonochem. 2025 Aug 6;120:107499. doi: 10.1016/j.ultsonch.2025.107499 (PMC12357160; doi:10.1016/j.ultsonch.2025.107499)
Supplement: Supplementary Data 4 [file mmc4.docx]

**Suppl. S4** Analysis of basic physicochemical properties of products across different groups.

**Note:** (ConR) represents the group of unprocessed green coffee beans. (NR) refers to naturally fermented green coffee beans. (CatR) denotes the group of civet-fermented green coffee beans. (CatIR) indicates the group of green coffee beans fermented through in vitro biomimetic methods. (ConC) represents the group of unprocessed roasted coffee beans. (NC) refers to naturally fermented roasted coffee beans. (CatC) denotes the group of civet-fermented roasted coffee beans. (CatIC) indicates the group of roasted coffee beans subjected to in vitro biomimetic fermentation.

Different letters within the same column indicate statistically significant differences (*P* < 0.05).

| Group | pH | Total acidity（meq/L） | TDS（mg/L） | Flavonoids（mg/L） | Polyphenols（mg/L） | Chlorogenic acid（mg/L） | Amino acids（mg/g） | Caffeine  (mg/g) |
| --- | --- | --- | --- | --- | --- | --- | --- | --- |
| ConR | 5.42±0.16^a^ | 22.00±3.19^c^ | 195.00±13.93^a^ | 14.01±0.79^a^ | 5.79±1.02^d^ | 9.43±0.81^a^ | 23.47±1.51^b^ | N/A |
| NR | 4.83±0.10^c^ | 39.14±1.01^a^ | 139.67±5.13^e^ | 10.58±1.14^c^ | 12.91±1.16^a^ | 7.46±0.58^b^ | 26.91±1.57^a^ | N/A |
| CatR | 4.73±0.41^c^ | 29.98±1.88^b^ | 191.00±11.14^b^ | 11.21±0.40^b^ | 10.85±0.90^b^ | 7.69±0.44^b^ | 20.65±1.44^c^ | N/A |
| CatIR | 4.94±0.31^b^ | 34.72±1.10^b^ | 157.33±25.10^c^ | 9.52±0.39^d^ | 11.47±0.47^b^ | 7.45±0.44^b^ | 31.46±1.69^a^ | N/A |
| ConC | 4.73±0.03^b^ | 27.33±0.58^b^ | 175.00±9.85^b^ | 7.76±0.34^e^ | 4.07±0.34^e^ | 9.26±0.26^a^ | 18.23±0.57^d^ | 13.40±0.09^a^ |
| NC | 4.74±0.03^b^ | 72.83±2.08^a^ | 128.33±7.64^d^ | 8.67±0.45^d^ | 5.89±0.31^d^ | 5.98±0.37^c^ | 17.59±0.63^d^ | 10.58±0.01^c^ |
| CatC | 4.72±0.03^b^ | 25.78±1.44^b^ | 152.33±14.01^c^ | 8.53±0.23^d^ | 6.01±0.30^d^ | 8.28±0.10^b^ | 16.85±0.57^d^ | 10.59±0.01^c^ |
| CatIC | 4.56±0.02^d^ | 25.56±0.42^b^ | 163.67±10.97^b^ | 7.10±0.38^f^ | 8.14±0.48^c^ | 7.31±0.18^b^ | 18.93±0.32^c^ | 13.51±0.01^a^ |
