# Supplementary material for: Ultrasound-assisted extraction and flavor quality assessment of in vitro biomimetically fermented Kopi Luwak
Source: Ultrason Sonochem. 2025 Aug 6;120:107499. doi: 10.1016/j.ultsonch.2025.107499 (PMC12357160; doi:10.1016/j.ultsonch.2025.107499)

**Suppl. S6** Significant enrichment analysis of metabolic pathways by in vitro biomimetic fermentation.

(A) Comparison between the in vitro biomimetic fermentation group and the untreated group of green coffee beans. (B) Comparison between the in vitro biomimetic fermentation group and the untreated group of roasted coffee beans.
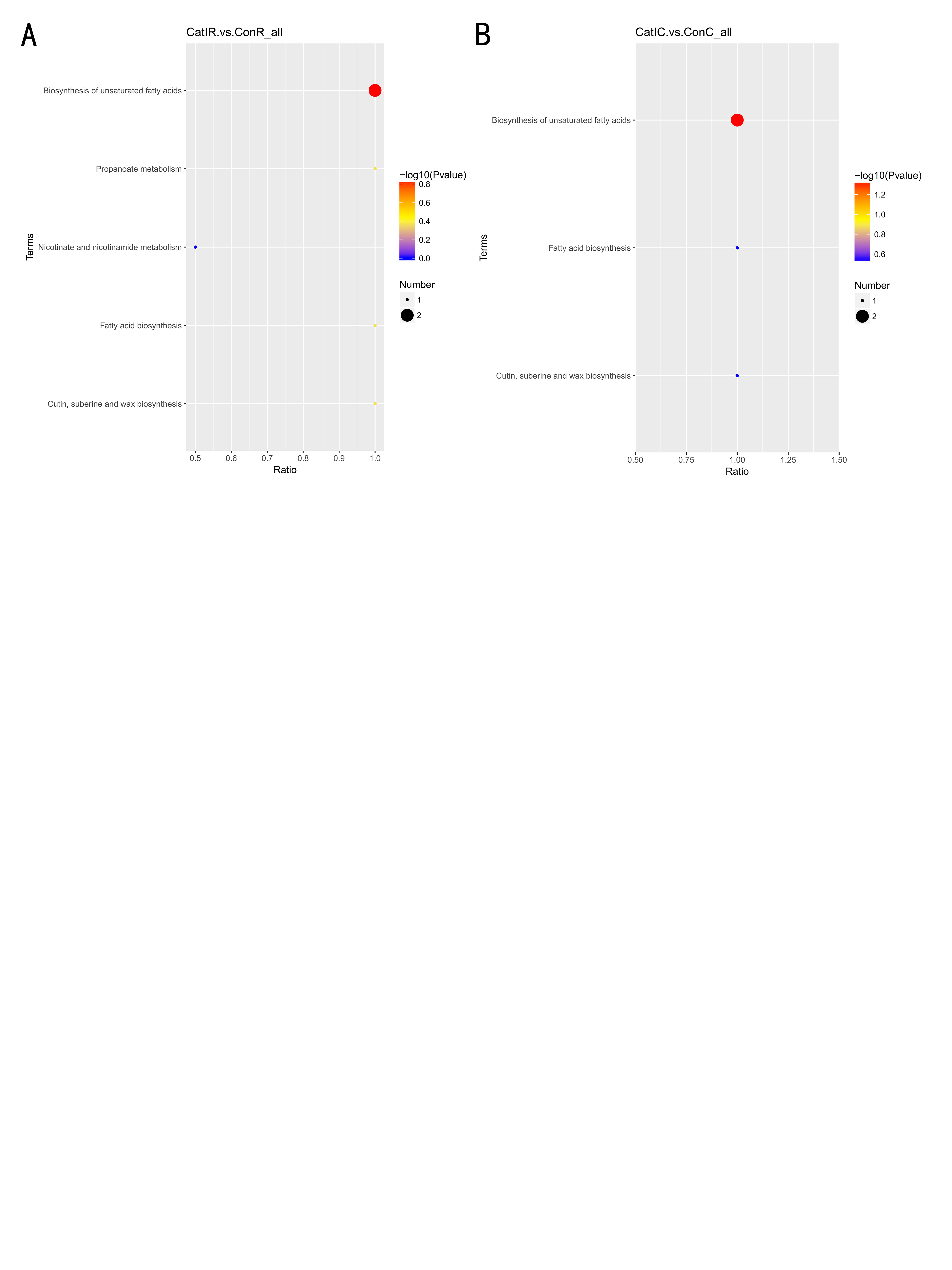


Unsaturated fatty acid biosynthesis pathway diagram and metabolite generation pathway analysis


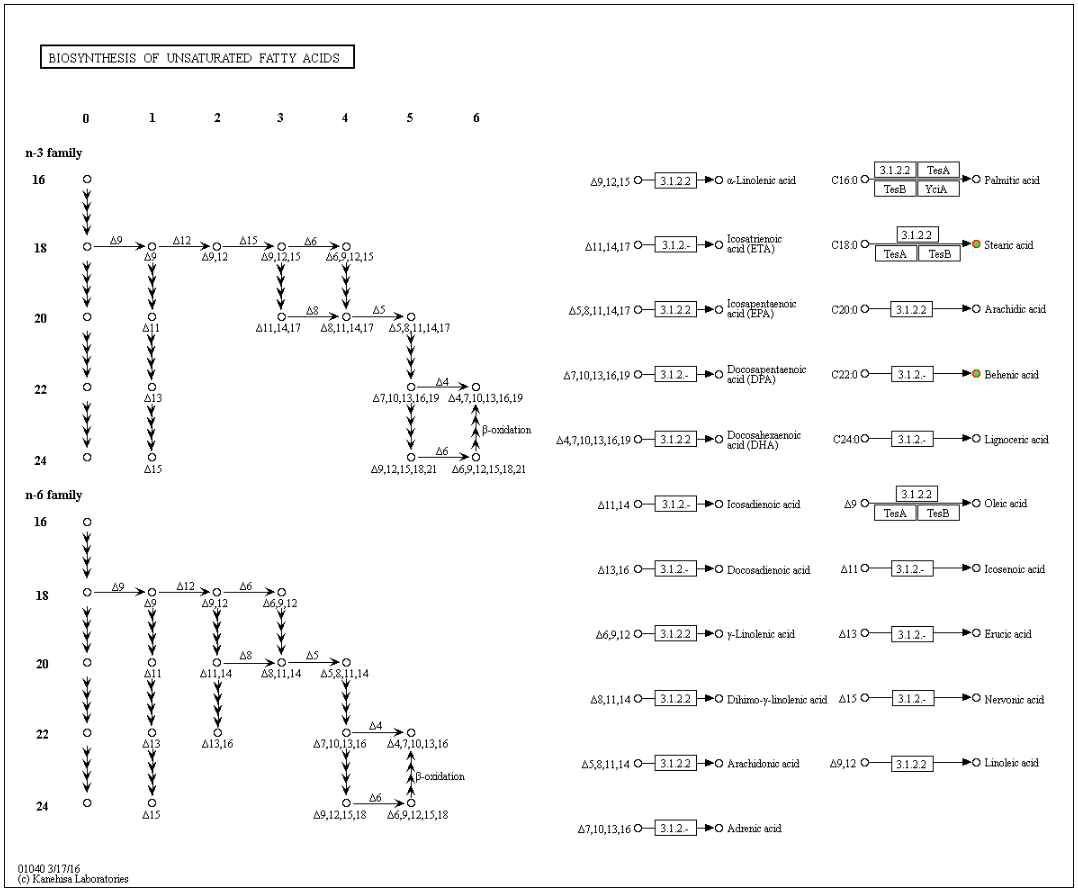

Supplement: Supplementary Data 6 [file mmc6.docx]
