# Supplementary figures and images for: Ultrasound-assisted extraction and flavor quality assessment of in vitro biomimetically fermented Kopi Luwak
Source: Ultrason Sonochem. 2025 Aug 6;120:107499. doi: 10.1016/j.ultsonch.2025.107499 (PMC12357160; doi:10.1016/j.ultsonch.2025.107499)

**Suppl. S7** Heatmap analysis of fermentation time-dependent metabolite changes.


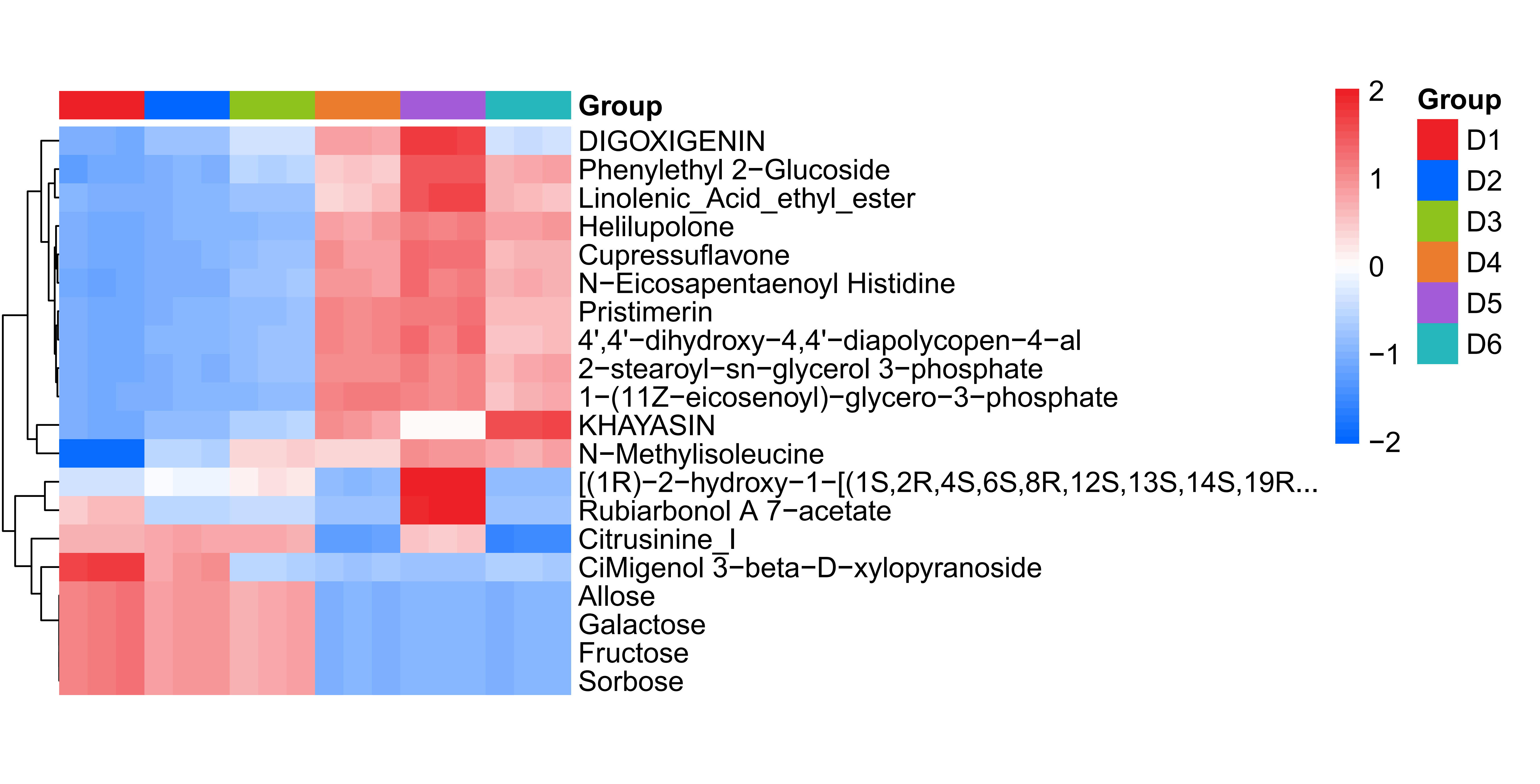

Supplement: Supplementary Data 7 [file mmc7.docx]

**Suppl. S11** Panorama of the main metabolic pathways of in vitro biomimetic fermented coffee.


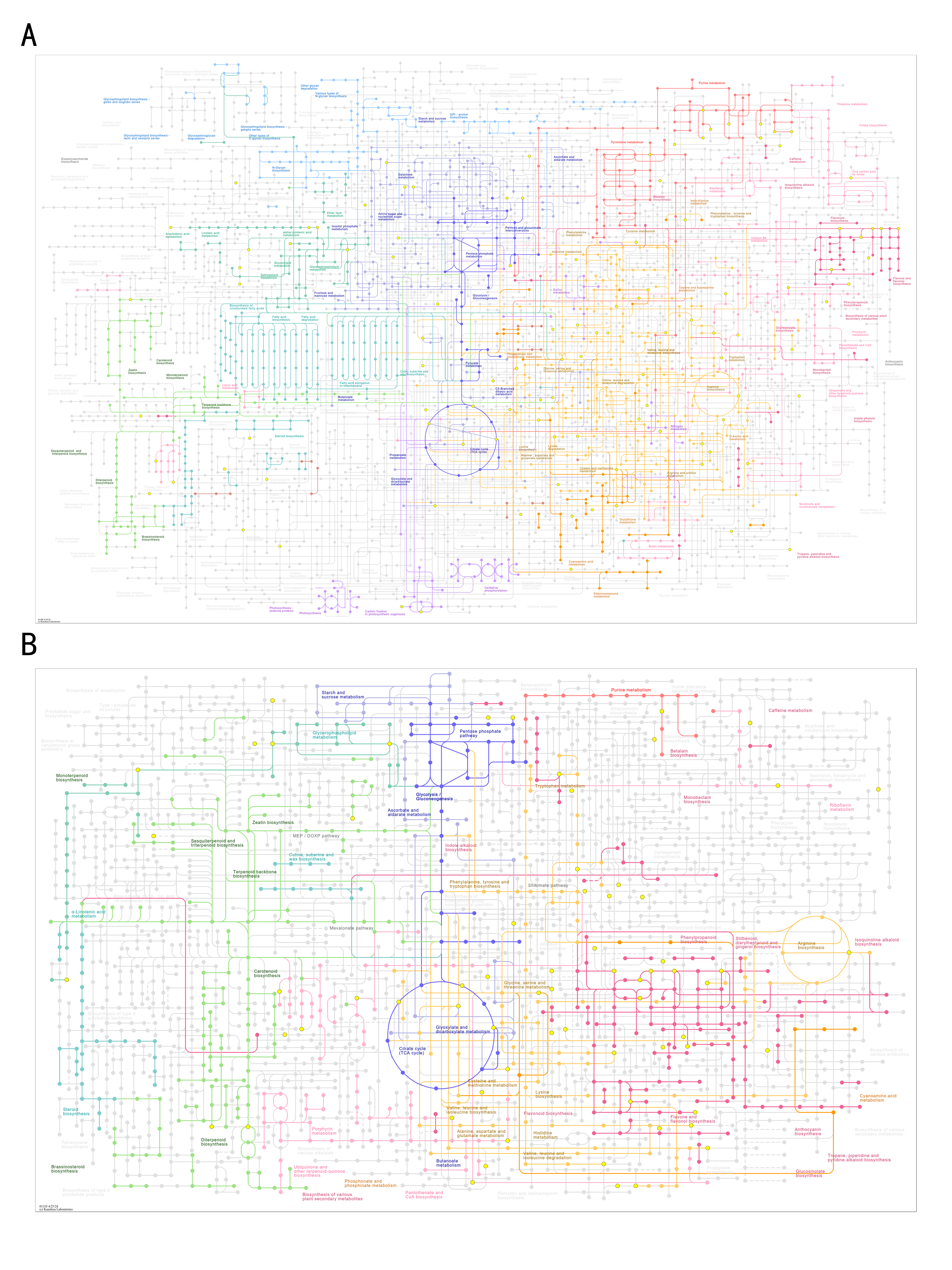

Supplement: Supplementary Data 11 [file mmc11.docx]
