# Supplementary material for: Ultrasound-assisted extraction and flavor quality assessment of in vitro biomimetically fermented Kopi Luwak
Source: Ultrason Sonochem. 2025 Aug 6;120:107499. doi: 10.1016/j.ultsonch.2025.107499 (PMC12357160; doi:10.1016/j.ultsonch.2025.107499)

**Suppl. S8** Heatmap analysis of fermentation time-dependent metabolite changes.

Note: (D) Different fermentation durations.


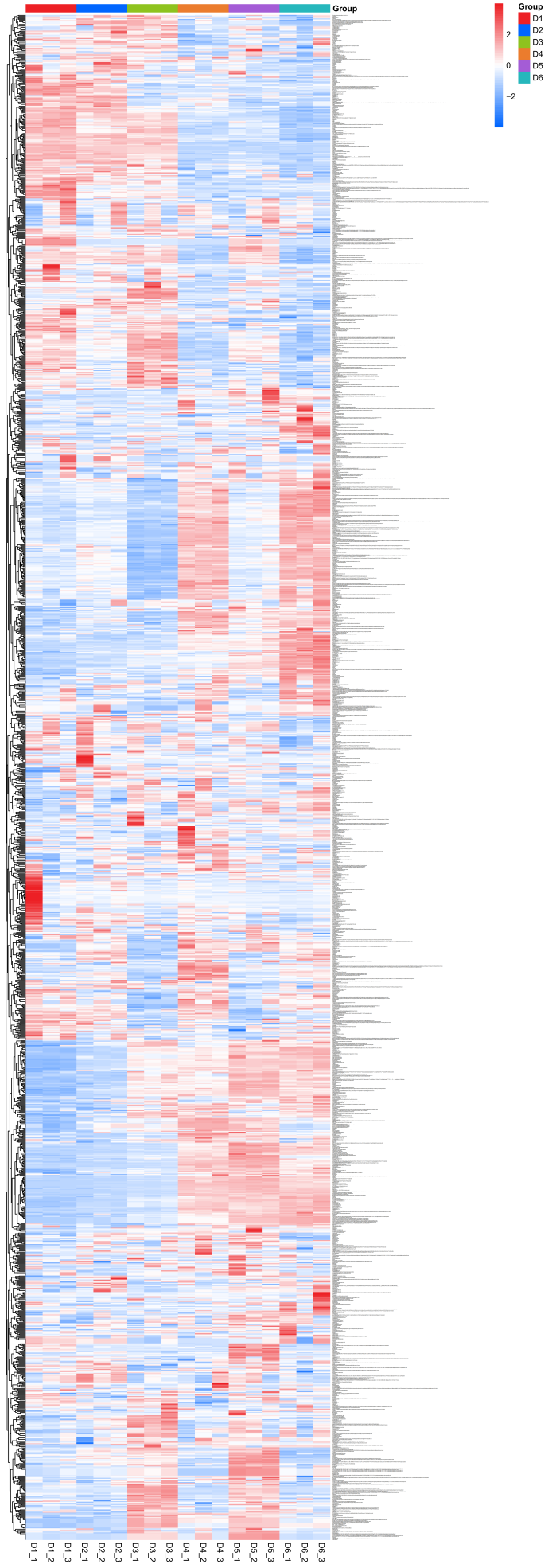

Supplement: Supplementary Data 8 [file mmc8.docx]
