# Supplementary material for: Ultrasound-assisted extraction and flavor quality assessment of in vitro biomimetically fermented Kopi Luwak
Source: Ultrason Sonochem. 2025 Aug 6;120:107499. doi: 10.1016/j.ultsonch.2025.107499 (PMC12357160; doi:10.1016/j.ultsonch.2025.107499)
Supplement: Supplementary Data 9 [file mmc9.docx]

**Suppl. S9** Network diagram of key metabolites in vitro biomimetic fermented coffee.
