# Supplementary material for: Ultrasound-assisted extraction and flavor quality assessment of in vitro biomimetically fermented Kopi Luwak
Source: Ultrason Sonochem. 2025 Aug 6;120:107499. doi: 10.1016/j.ultsonch.2025.107499 (PMC12357160; doi:10.1016/j.ultsonch.2025.107499)

**Suppl. S10** KEGG pathway enrichment and metabolic network analysis in vitro biomimetic fermentation process.

(A) Bar chart of KEGG pathway enrichment analysis. (B) Significance tree diagram of KEGG pathway enrichment. (C) Metabolic network relationship diagram.
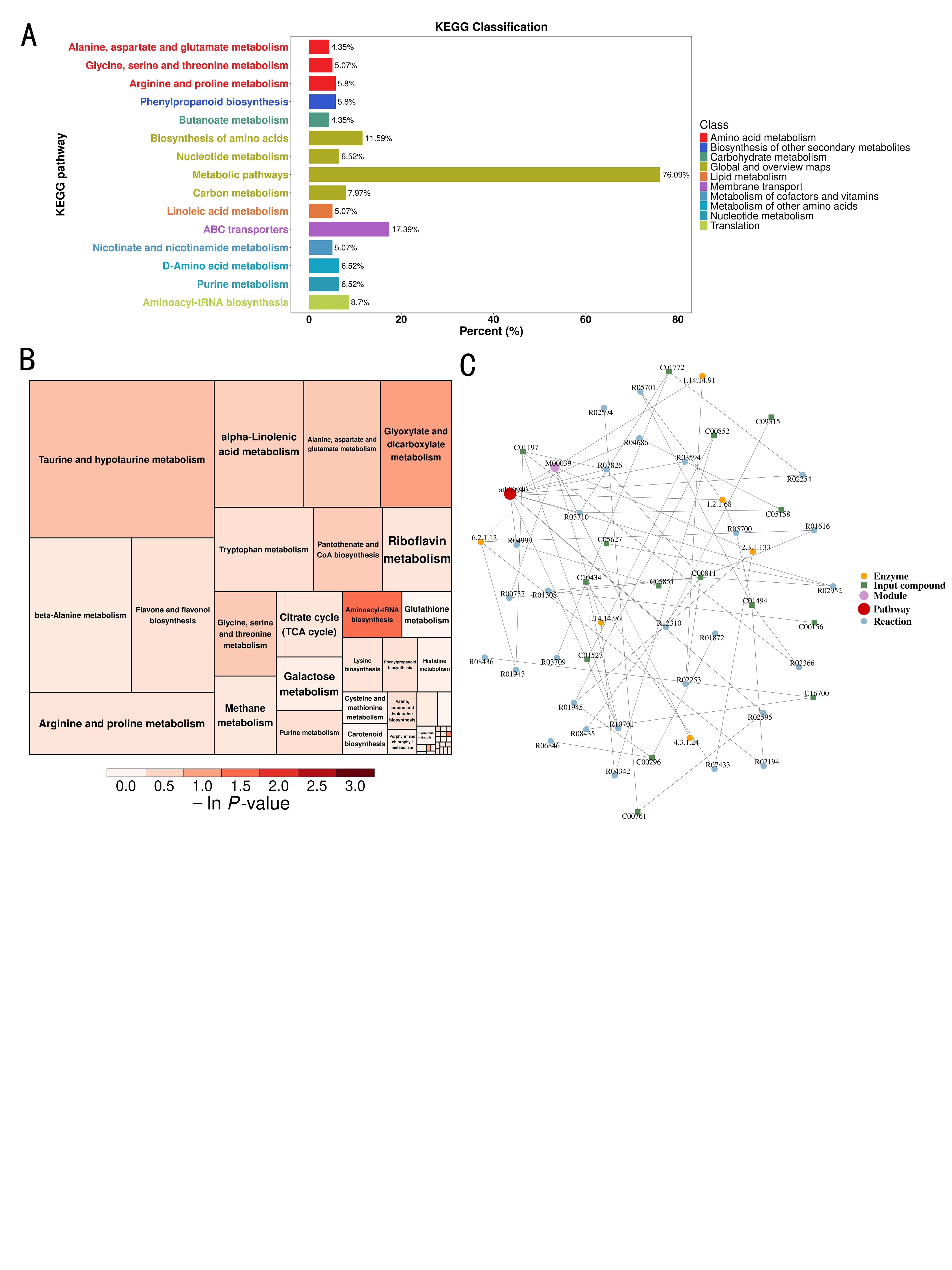

Supplement: Supplementary Data 10 [file mmc10.docx]
