# Supplementary material for: Ultrasound-assisted extraction and flavor quality assessment of in vitro biomimetically fermented Kopi Luwak
Source: Ultrason Sonochem. 2025 Aug 6;120:107499. doi: 10.1016/j.ultsonch.2025.107499 (PMC12357160; doi:10.1016/j.ultsonch.2025.107499)

**Suppl. S12** (A) Key physicochemical parameters and SCA scores under enzyme-assisted shortened fermentation. (B) Cost-sensitivity analysis of inoculum volume versus total processing cost (1-t wet beans).


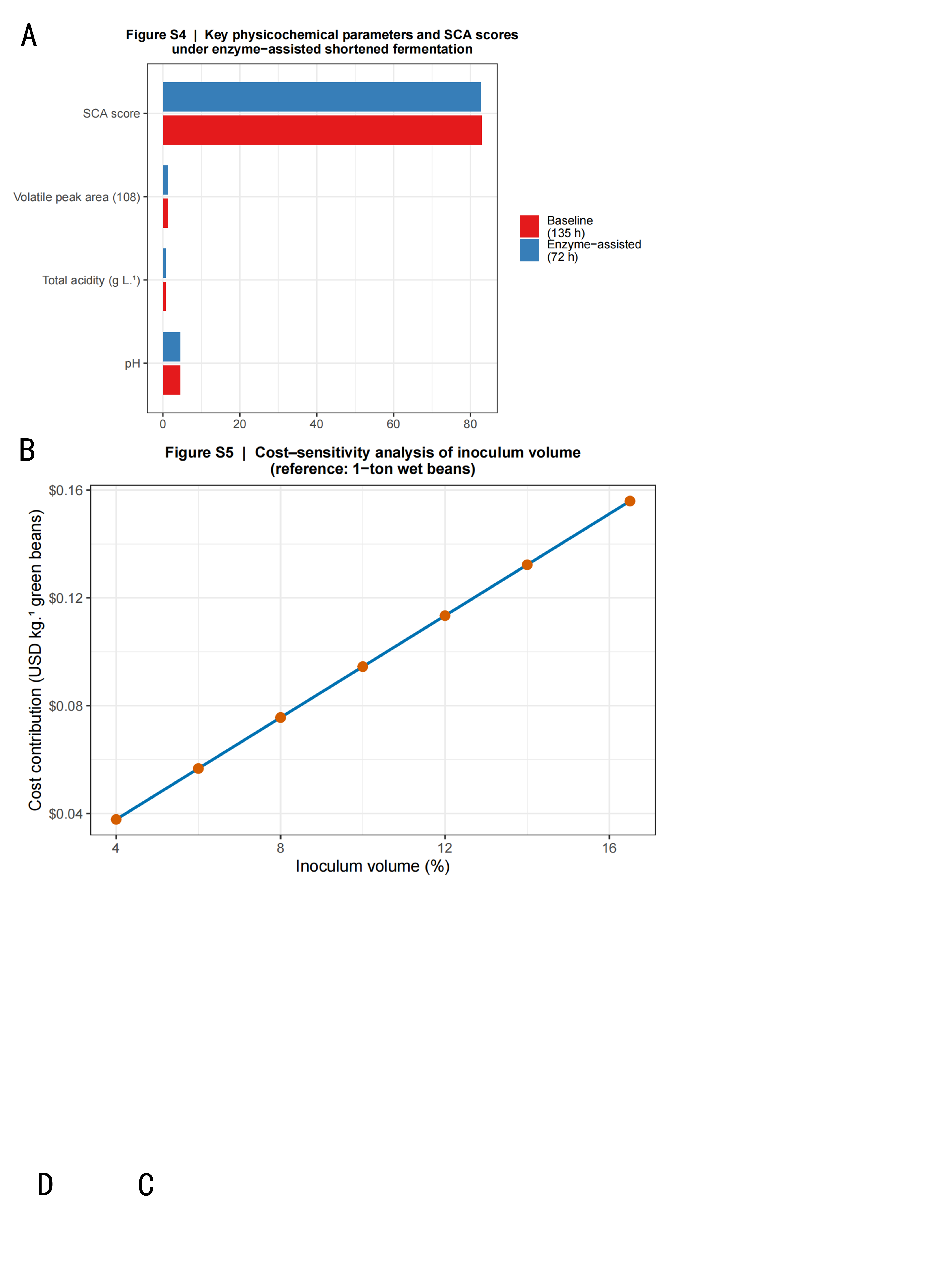

Supplement: Supplementary Data 12 [file mmc12.docx]
