# Supplementary material for: Ultrasound-assisted extraction and flavor quality assessment of in vitro biomimetically fermented Kopi Luwak
Source: Ultrason Sonochem. 2025 Aug 6;120:107499. doi: 10.1016/j.ultsonch.2025.107499 (PMC12357160; doi:10.1016/j.ultsonch.2025.107499)

**Suppl. S13** (A)Sensory_Overall_Distribution；(B) Kappa_ByAttribute

Note:Con：club members；Pro：CQI-licensed Q-Graders


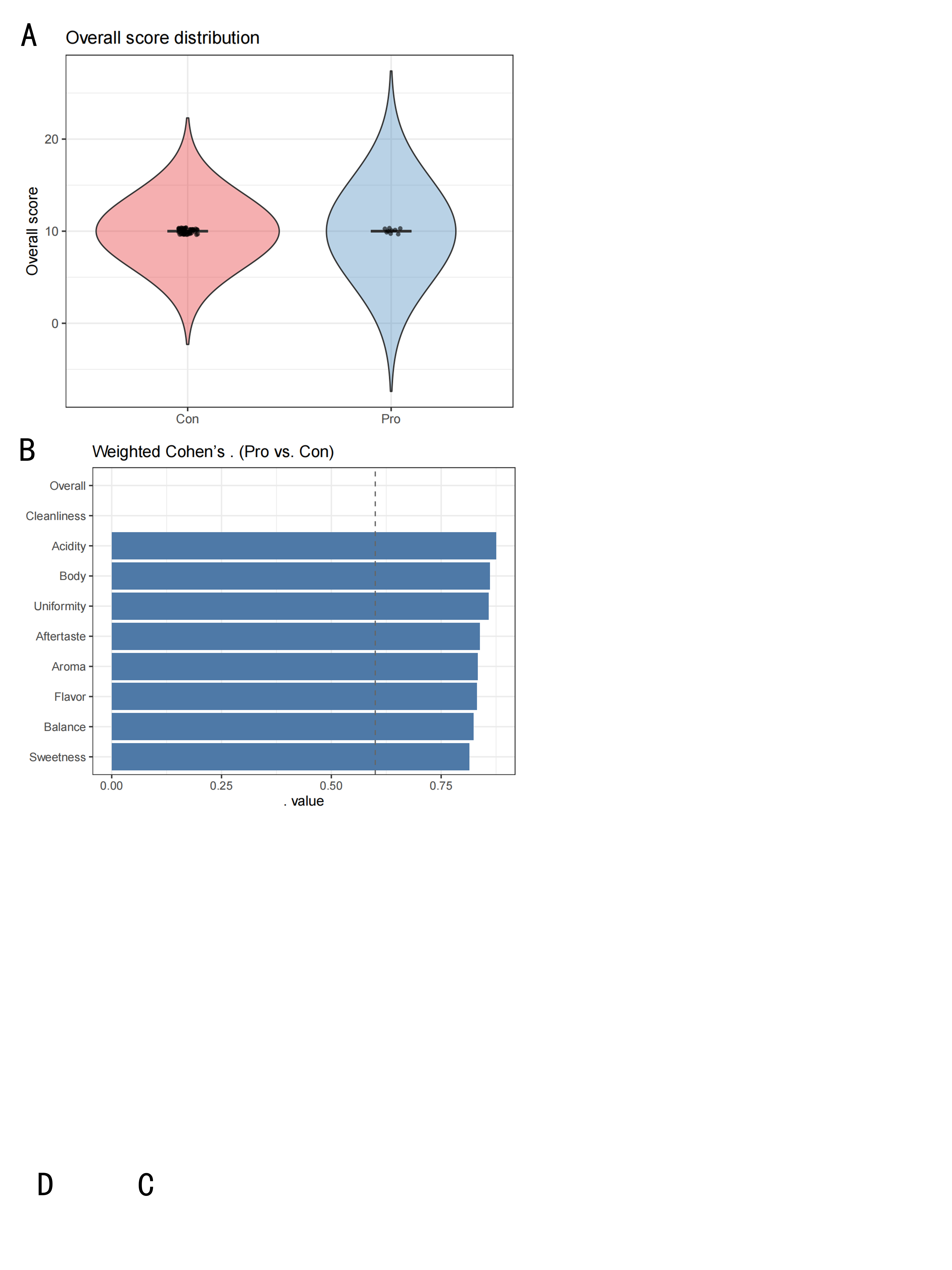

Supplement: Supplementary Data 13 [file mmc13.docx]
