# Supplementary material for: Ultrasound-assisted extraction and flavor quality assessment of in vitro biomimetically fermented Kopi Luwak
Source: Ultrason Sonochem. 2025 Aug 6;120:107499. doi: 10.1016/j.ultsonch.2025.107499 (PMC12357160; doi:10.1016/j.ultsonch.2025.107499)
Supplement: Supplementary Data 14 [file mmc14.docx]

**Suppl. S14** Dynamic changes of extracellular esterase and protease activities during fermentation (mean ± SD, n = 3).

| Time (h) | Esterase (U g⁻¹) | Protease (U g⁻¹) | Esterase (U g⁻¹) | Protease (U g⁻¹) |
| --- | --- | --- | --- | --- |
|  | CatIC | CatIC | CatC | CatC |
| 0 | 12.4 ± 0.7 | 8.6 ± 0.5 | 12.1 ± 0.6 | 8.3 ± 0.4 |
| 24 | 23.8 ± 1.1 | 15.3 ± 0.9 | 19.2 ± 0.9 | 12.5 ± 0.6 |
| 72 | 38.4 ± 1.4 | 26.4 ± 1.2 | 24.3 ± 1.2 | 17.4 ± 1.1 |
| 135 | 45.7 ± 1.8 | 33.2 ± 1.3 | 27.9 ± 1.3 | 19.8 ± 1.2 |
