# Supplementary material for: Ultrasound-assisted extraction and flavor quality assessment of in vitro biomimetically fermented Kopi Luwak
Source: Ultrason Sonochem. 2025 Aug 6;120:107499. doi: 10.1016/j.ultsonch.2025.107499 (PMC12357160; doi:10.1016/j.ultsonch.2025.107499)

**Suppl. S15** GA-ANN Network topology diagram

Note：Input layer (X₁–X₄)

• X₁ – Starter inoculum concentration (%)

• X₂ – Initial fermentation pH

• X₃ – Fermentation temperature (℃)

• X₄ – Fermentation duration (h)

Each input node is fully connected to every neuron in the hidden layer. Individual connection weights are denoted wᵢⱼ; the multicoloured lines simply distinguish different pathways.

Hidden layer (H₁–H₅)

• Five neurons (H₁–H₅); each carries its own bias term bᵢ (grey dot).

• The net input to each neuron S = Σ(wᵢⱼ · Xⱼ) + bᵢ is passed through a ReLU activation function before being forwarded to the next layer.

Output layer (Y)

• Single node Y that yields the predicted SCA cupping score.

• Connections from the hidden neurons to Y have weights Wₖ and a bias bᵧ.

Predicted score: Ŷ = Σ(Wₖ · Hₖ) + bᵧ (identity activation).

Training and optimisation

• Initial weights are generated by a genetic algorithm (GA), providing a global search of the weight space.

• The network is subsequently fine-tuned via back-propagation (BP) to minimise the mean-squared error (MSE) between Ŷ and the experimental SCA scores.

• The final model converged after 587 GA generations followed by 200 BP epochs.


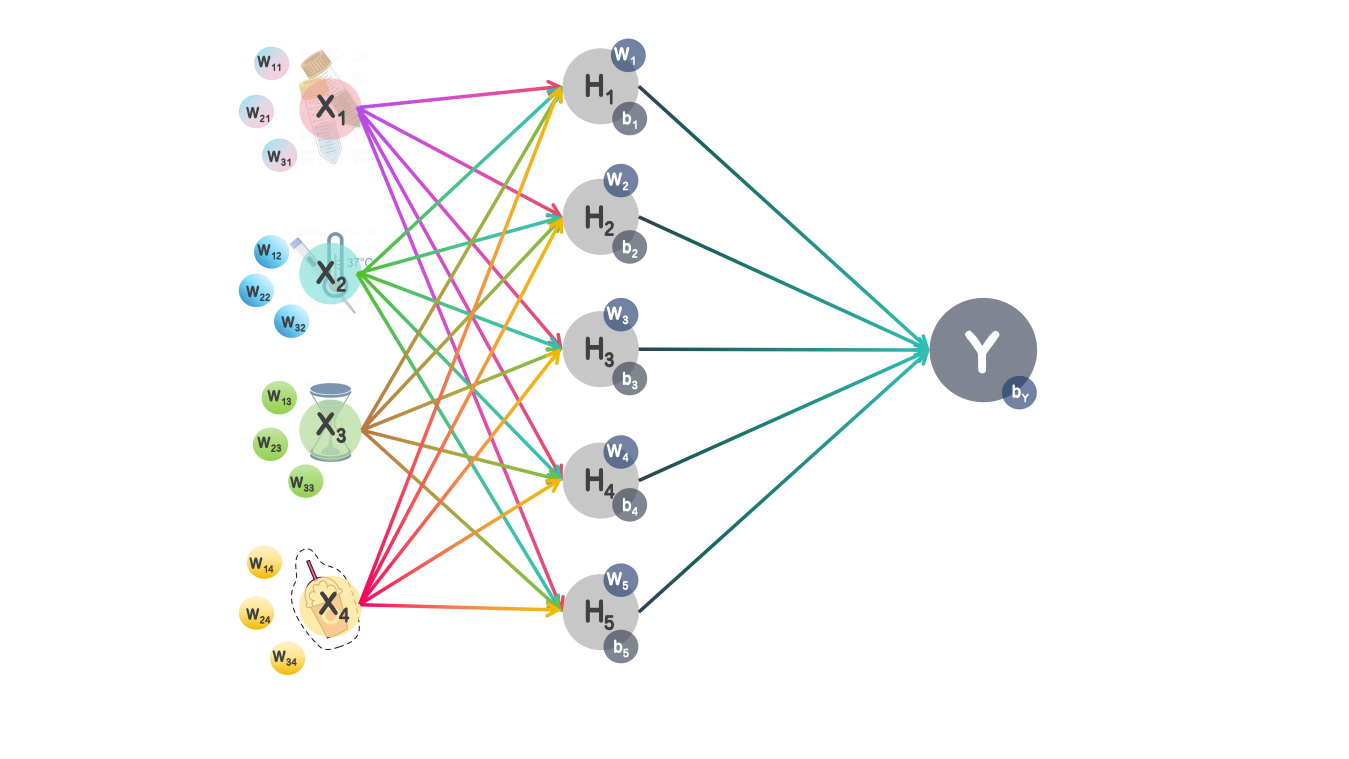

Supplement: Supplementary Data 15 [file mmc15.docx]
