# Supplementary material for: Ultrasound-assisted extraction and flavor quality assessment of in vitro biomimetically fermented Kopi Luwak
Source: Ultrason Sonochem. 2025 Aug 6;120:107499. doi: 10.1016/j.ultsonch.2025.107499 (PMC12357160; doi:10.1016/j.ultsonch.2025.107499)

**Suppl. S16** (A) Sensory_Radar;(B) Volcano

Note:B) Volcano plot showing differential volatile metabolites between CatIC and CatC; red, up-regulated; blue, down-regulated; threshold: |log₂FC| > 1 & FDR < 0.05.


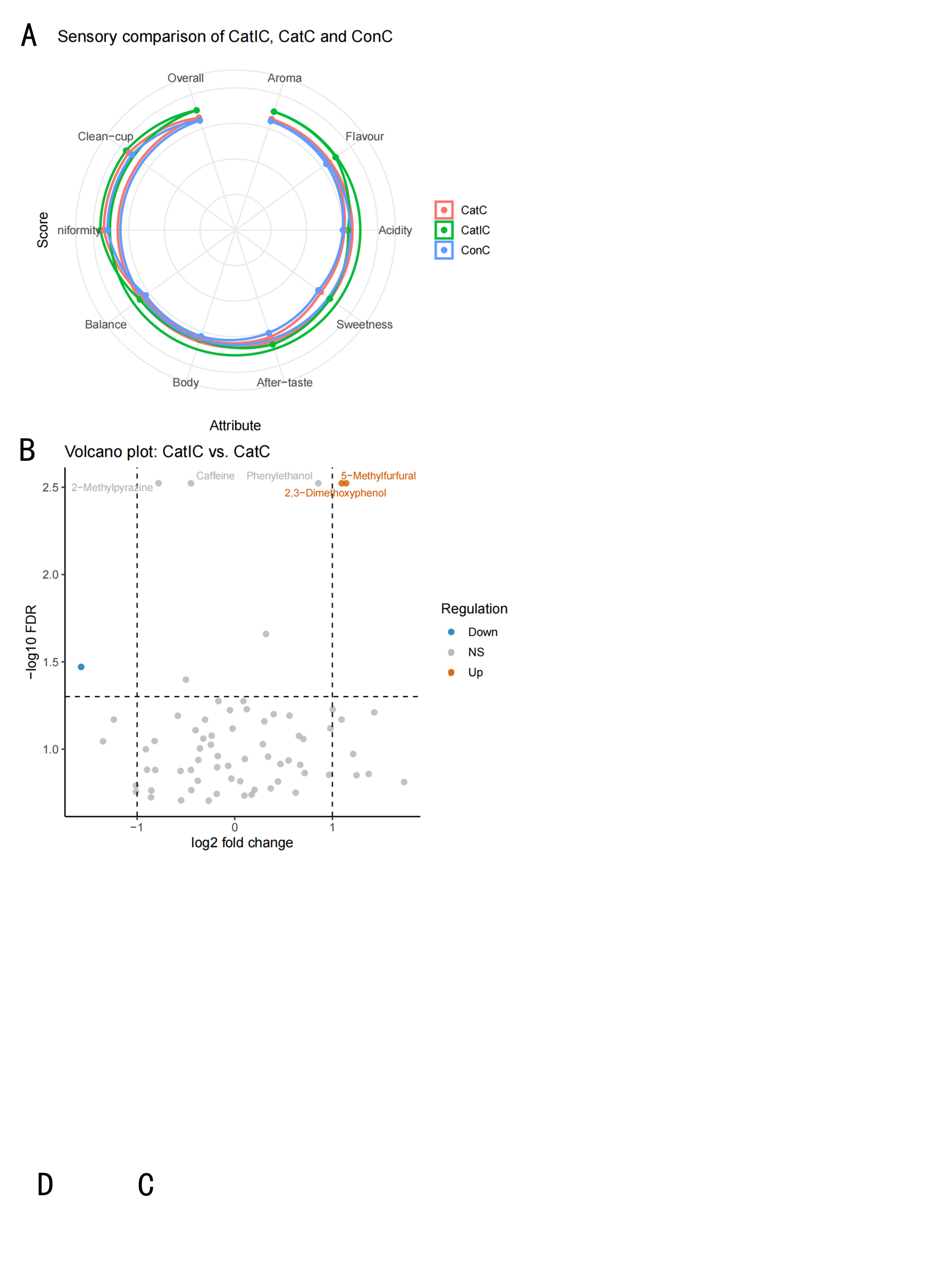

Supplement: Supplementary Data 16 [file mmc16.docx]
