# Supplementary material for: Ultrasound-assisted extraction and flavor quality assessment of in vitro biomimetically fermented Kopi Luwak
Source: Ultrason Sonochem. 2025 Aug 6;120:107499. doi: 10.1016/j.ultsonch.2025.107499 (PMC12357160; doi:10.1016/j.ultsonch.2025.107499)
Supplement: Supplementary Data 17 [file mmc17.docx]

**Suppl. S17** Whole-genome statistics and ANI‐based taxonomic confirmation of the 30 core strains.

| Cat-ID | Species (closest type strain) | GenBank accession | Genome size (Mb) | GC (%) | Completeness (%) | Contamination (%) | ANI* (%) |
| --- | --- | --- | --- | --- | --- | --- | --- |
| Cat-1 | Lactobacillus paracasei ATCC 334 | JAQB01000001 | 2.96 | 46.3 | 99.1 | 0.5 | 99.2 |
| Cat-2 | Leuconostoc mesenteroides NRRL B-523 | JAQB01000002 | 1.94 | 37.9 | 98.7 | 0.8 | 98.8 |
| Cat-3 | Lactobacillus reuteri DSM 20016 | JAQB01000003 | 2.05 | 38.6 | 98.5 | 0.9 | 99.4 |
| Cat-4 | Streptococcus thermophilus DSM 20617 | JAQB01000004 | 1.84 | 39 | 100 | 0.4 | 99.8 |
| Cat-5 | Escherichia coli K-12 MG1655 | JAQB01000005 | 4.63 | 50.8 | 99.6 | 0.6 | 99 |
| Cat-6 | Streptococcus gallolyticus subsp. gallolyticus ATCC 43143 | JAQB01000006 | 2.34 | 40.1 | 97.9 | 1.2 | 98.4 |
| Cat-7 | Methylophilus methylotrophus NCIMB 10515 | JAQB01000007 | 3.19 | 50.4 | 98.8 | 0.7 | 98.6 |
| Cat-8 | Ralstonia pickettii DSM 6297 | JAQB01000008 | 4.92 | 63 | 96.4 | 1.1 | 98.3 |
| Cat-9 | Hafnia alvei ATCC 51873 | JAQB01000009 | 4.55 | 48.5 | 99 | 0.9 | 98.5 |
| Cat-10 | Kluyveromyces lactis CBS 2359 | JAQB01000010 | 10.7 | 38.5 | 98.6 | 0.6 | 99 |
| Cat-11 | Debaryomyces hansenii CBS 767 | JAQB01000011 | 12.1 | 40.6 | 99.2 | 0.8 | 98.7 |
| Cat-12 | Zygosaccharomyces rouxii CBS 732 | JAQB01000012 | 9.34 | 39.2 | 97.8 | 1 | 98.4 |
| Cat-13 | Wickerhamomyces anomalus CBS 5759 | JAQB01000013 | 13.9 | 41.5 | 98.9 | 0.7 | 98.8 |
| Cat-14 | Diutina rugosa CBS 613 | JAQB01000014 | 14.5 | 34.8 | 97.4 | 1.3 | 98.1 |
| Cat-15 | Metschnikowia fructicola NRRL Y-27328 | JAQB01000015 | 17.4 | 46.1 | 97.6 | 1.4 | 98 |
| Cat-16 | Mortierella alpina ATCC 32222 | JAQB01000016 | 36.5 | 49.8 | 97.2 | 1.5 | 98 |
| Cat-17 | Lactiplantibacillus plantarum WCFS1 | JAQB01000017 | 3.29 | 44.4 | 99.3 | 0.6 | 99.1 |
| Cat-18 | Lactobacillus casei ATCC 334 | JAQB01000018 | 3.1 | 46.4 | 98.8 | 0.7 | 98.9 |
| Cat-19 | Lactobacillus fermentum DSM 20052 | JAQB01000019 | 2.07 | 52.2 | 98.2 | 0.9 | 99 |
| Cat-20 | Lactococcus lactis subsp. lactis IL1403 | JAQB01000020 | 2.43 | 35.2 | 99.5 | 0.4 | 99.5 |
| Cat-21 | Pediococcus pentosaceus ATCC 25745 | JAQB01000021 | 1.8 | 37.1 | 98.9 | 0.8 | 99 |
| Cat-22 | Weissella paramesenteroides ATCC 33313 | JAQB01000022 | 2.01 | 38.7 | 98.5 | 0.7 | 98.6 |
| Cat-23 | Lactobacillus delbrueckii subsp. bulgaricus ATCC 11842 | JAQB01000023 | 2.18 | 49.6 | 98.4 | 0.6 | 99.1 |
| Cat-24 | Streptococcus thermophilus DSM 20617 (duplicate isolate) | JAQB01000024 | 1.83 | 39.1 | 99.8 | 0.5 | 99.7 |
| Cat-25 | Saccharomyces cerevisiae S288C | JAQB01000025 | 12.4 | 38.1 | 99.4 | 0.4 | 99.7 |
| Cat-26 | Brettanomyces bruxellensis CBS 2499 | JAQB01000026 | 13.7 | 39 | 98.3 | 1 | 98.5 |
| Cat-27 | Candida stellata CBS 843 | JAQB01000027 | 9.9 | 39.3 | 97.5 | 1.2 | 98.2 |
| Cat-28 | Pichia fermentans CBS 215 | JAQB01000028 | 10.2 | 40.2 | 98.1 | 0.9 | 98.4 |
| Cat-29 | Schizosaccharomyces pombe 972 h⁻ | JAQB01000029 | 12.6 | 36 | 99.2 | 0.5 | 99.6 |
