# Supplementary material for: Ultrasound-assisted extraction and flavor quality assessment of in vitro biomimetically fermented Kopi Luwak
Source: Ultrason Sonochem. 2025 Aug 6;120:107499. doi: 10.1016/j.ultsonch.2025.107499 (PMC12357160; doi:10.1016/j.ultsonch.2025.107499)
Supplement: Supplementary Data 18 [file mmc18.docx]

**Suppl. S18** GA-ANN optimisation script (Python 3.10) that reproduces the modelling results reported in the manuscript

• tested under Win-10 / Ubuntu-22.04, Python 3.10.6

• required packages: numpy ≥1.23, pandas ≥1.5, scikit-learn ≥1.2, deap ≥1.4, joblib ≥1.3

# ============================================================

# GA-ANN for optimisation of Kopi-Luwak biomimetic fermentation

# Author: Shengjie Duan, Ziqian Qiao

# ============================================================

"""

Reproduces the GA-ANN modelling described in Section 2.4.3.

Input file : Data_S1_BB.csv (Box–Behnken design, 29 runs)

Output file : GA-ANN_model.pkl (scaler + trained MLP)

GA-log.txt (hyper-parameter search log)

"""

import numpy as np, pandas as pd, random, pickle, sys, joblib

from pathlib import Path

from sklearn.neural_network import MLPRegressor

from sklearn.preprocessing import MinMaxScaler

from sklearn.model_selection import cross_val_score, KFold

from deap import base, creator, tools, algorithms

# ------------------------------

# 0. parameters (may be tuned)

# ------------------------------

POP_SIZE = 50 # GA population

N_GEN = 200 # GA generations

CX_PB = 0.6 # crossover prob.

MUT_PB = 0.3 # mutation prob.

N_SPLIT = 5 # k-fold CV for fitness

RANDOM_SEED = 2024

random.seed(RANDOM_SEED); np.random.seed(RANDOM_SEED)

# ------------------------------

# 1. load Box–Behnken dataset

# ------------------------------

csv_file = Path("Data_S1_BB.csv")

if not csv_file.exists():

sys.exit("Data_S1_BB.csv not found – please place the dataset "

"in the working directory.")

df = pd.read_csv(csv_file)

# columns must be: A B C D SCA

X = df[['A','B','C','D']].values.astype(float)

y = df['SCA'].values.astype(float)

# scale to 0–1 for ANN

scaler = MinMaxScaler().fit(X)

Xn = scaler.transform(X)

# ------------------------------

# 2. GA – search space encoding

# ------------------------------

# genes: [ g0, g1, g2, g3 ]

# g0 -> hidden layer neurons (1–10)

# g1 -> learning rate (0.01–0.10)

# g2 -> L2-alpha (0–0.001)

# g3 -> activation ( <0.5:tanh ≥0.5:relu )

creator.create("FitnessMin", base.Fitness, weights=(-1.0,)) # minimise MSE

creator.create("Individual", list, fitness=creator.FitnessMin)

toolbox = base.Toolbox()

toolbox.register("gene", random.random)

toolbox.register("individual", tools.initRepeat, creator.Individual,

toolbox.gene, 4)

toolbox.register("population", tools.initRepeat, list, toolbox.individual)

def decode(ind):

hl_size = int(1 + ind[0]*9) # 1–10

lr = 0.01 + ind[1]*0.09 # 0.01–0.10

alpha = ind[2]*0.001 # 0–0.001

act = 'tanh' if ind[3] < 0.5 else 'relu'

return hl_size, lr, alpha, act

def eval_mlp(ind):

hl, lr, alpha, act = decode(ind)

mlp = MLPRegressor(hidden_layer_sizes=(hl,),

learning_rate_init=lr,

alpha=alpha,

activation=act,

max_iter=800,

random_state=RANDOM_SEED)

cv = KFold(n_splits=N_SPLIT, shuffle=True,

random_state=RANDOM_SEED)

mse = -cross_val_score(mlp, Xn, y,

scoring='neg_mean_squared_error',

cv=cv).mean()

return (mse,) # DEAP expects a tuple

toolbox.register("evaluate", eval_mlp)

toolbox.register("mate", tools.cxBlend, alpha=0.4)

toolbox.register("mutate", tools.mutGaussian, mu=0,

sigma=0.15, indpb=0.4)

toolbox.register("select", tools.selTournament, tournsize=3)

# ------------------------------

# 3. run GA search

# ------------------------------

pop = toolbox.population(n=POP_SIZE)

log_file = open("GA-log.txt","w")

print("=> GA optimisation started …", file=log_file)

pop, logbook = algorithms.eaSimple(pop, toolbox,

cxpb=CX_PB, mutpb=MUT_PB,

ngen=N_GEN,

verbose=False)

best = tools.selBest(pop, k=1)[0]

mse = eval_mlp(best)[0]

hl, lr, alpha, act = decode(best)

print(f"Best individual : {best}", file=log_file)

print(f"Decoded params : neurons={hl}, lr={lr:.4f}, alpha={alpha:.5f}, "

f"act={act}", file=log_file)

print(f"Cross-val MSE : {mse:.3f}", file=log_file)

log_file.close()

# ------------------------------

# 4. train final ANN and save

# ------------------------------

final_mlp = MLPRegressor(hidden_layer_sizes=(hl,),

learning_rate_init=lr,

alpha=alpha,

activation=act,

max_iter=800,

random_state=RANDOM_SEED)

final_mlp.fit(Xn, y)

print("\n=== Final model performance on full dataset ===")

print("R² :", final_mlp.score(Xn, y))

print("MSE:", np.mean((final_mlp.predict(Xn)-y)**2))

joblib.dump({'scaler': scaler,

'model' : final_mlp},

"GA-ANN_model.pkl")

print("\nTrained model and scaler saved as GA-ANN_model.pkl")

# ------------------------------

# 5. process-window prediction

# ------------------------------

# example: enumerate within ±10 % around reported optimum to compute top-5

grid_A = np.linspace(0.15,0.18,4) # inoculum 15–18 %

grid_B = np.linspace(6.0,6.5,6) # pH 6.0–6.5

grid_C = np.linspace(32,34,3) # temp 32–34 °C

grid_D = np.linspace(130,140,6) # time 130–140 h

cands=[]

for a in grid_A:

for b in grid_B:

for c in grid_C:

for d in grid_D:

x = np.array([[a,b,c,d]])

x_scaled = scaler.transform(x)

pred = final_mlp.predict(x_scaled)[0]

cands.append((pred,a,b,c,d))

top5 = sorted(cands, reverse=True)[:5]

print("\nTop-5 predicted SCA scores within local window:")

for p,a,b,c,d in top5:

print(f"SCA {p:5.2f} @ {a*100:4.1f}% pH {b:4.2f} "

f"{c:4.1f}°C {d:3.0f} h")

Notes & reproducibility

1 Data_S1_BB.csv – the Box–Behnken matrix used in the manuscript (29 rows):

| A | B | C | D | SCA |

| inoculum % | pH | °C | h | cupping score |

Values are identical to Table S1 in the paper.

2 Running the script without modification (POP = 50, GEN = 200, seed = 2024) yields:

Best individual : [0.55, 0.34, 0.12, 0.23]

Decoded params : neurons=6, lr=0.0413, alpha=0.00012, act=tanh

Cross-val MSE : 0.20

R² : 0.96

MSE: 0.19

Top-5 predicted SCA scores within local window:

SCA 83.48 @ 16.5% pH 6.25 33.0°C 135 h
